# Can auxiliary indicators improve COVID-19 forecasting and hotspot prediction?

**DOI:** 10.1073/pnas.2111453118

**Published:** 2021-12-13

**Authors:** Daniel J. McDonald, Jacob Bien, Alden Green, Addison J. Hu, Nat DeFries, Sangwon Hyun, Natalia L. Oliveira, James Sharpnack, Jingjing Tang, Robert Tibshirani, Valérie Ventura, Larry Wasserman, Ryan J. Tibshirani

**Affiliations:** ^a^Department of Statistics, University of British Columbia, Vancouver, BC, Canada V6T 1Z4;; ^b^Department of Data Sciences and Operations, University of Southern California, Los Angeles, CA 90089;; ^c^Department of Statistics & Data Science, Carnegie Mellon University, Pittsburgh, PA 15213;; ^d^Machine Learning Department, Carnegie Mellon University, Pittsburgh, PA 15213;; ^e^Department of Statistics, University of California, Davis, CA 95616;; ^f^Computational Biology Department, Carnegie Mellon University, Pittsburgh, PA 15213;; ^g^Department of Statistics, Stanford University, Stanford, CA 94305;; ^h^Department of Biomedical Data Science, Stanford University, Stanford, CA 94305

**Keywords:** COVID-19, forecasting, hotspot prediction, time series, digital surveillance

## Abstract

Short-term forecasts of traditional streams from public health reporting (such as cases, hospitalizations, and deaths) are a key input to public health decision-making during a pandemic. Since early 2020, our research group has worked with data partners to collect, curate, and make publicly available numerous real-time COVID-19 indicators, providing multiple views of pandemic activity in the United States. This paper studies the utility of five such indicators—derived from deidentified medical insurance claims, self-reported symptoms from online surveys, and COVID-related Google search activity—from a forecasting perspective. For each indicator, we ask whether its inclusion in an autoregressive (AR) model leads to improved predictive accuracy relative to the same model excluding it. Such an AR model, without external features, is already competitive with many top COVID-19 forecasting models in use today. Our analysis reveals that 1) inclusion of each of these five indicators improves on the overall predictive accuracy of the AR model; 2) predictive gains are in general most pronounced during times in which COVID cases are trending in “flat” or “down” directions; and 3) one indicator, based on Google searches, seems to be particularly helpful during “up” trends.

## Significance

Validated forecasting methodology should be a vital element in the public health response to any fast-moving epidemic or pandemic. A widely used model for predicting the future spread of a temporal process is an autoregressive (AR) model. While basic, such an AR model (properly trained) is already competitive with the top models in operational use for COVID-19 forecasting. In this paper, we exhibit five auxiliary indicators—based on deidentified medical insurance claims, self-reported symptoms via online surveys, and COVID-related Google searches—that further improve the predictive accuracy of an AR model in COVID-19 forecasting. The most substantial gains appear to be in quiescent times; but the Google search indicator appears to also offer improvements during upswings in pandemic activity.

Tracking and forecasting indicators from public health reporting streams—such as confirmed cases and deaths in the COVID-19 pandemic—are crucial for understanding disease spread, correctly formulating public policy responses, and rationally planning future public health resource needs. A companion paper (1) describes our research group’s efforts, beginning in April 2020, in curating and maintaining a database of real-time indicators that track COVID-19 activity and other relevant phenomena. The signals (a term we use synonymously with “indicators”) in this database are accessible through the COVIDcast Application Programming Interface (API) (2), as well as associated R (3) and Python (4) packages, for convenient data fetching and processing. In the current paper, we quantify the utility provided by a core set of these indicators for two fundamental prediction tasks: probabilistic forecasting of COVID-19 case rates and prediction of future COVID-19 case hotspots (defined by the event that a relative increase in COVID-19 cases exceeds a certain threshold).

At the outset, we should be clear that our intent in this paper is not to provide an authoritative take on cutting-edge COVID-19 forecasting methods. Similarly, some authors, e.g., ref. 5, have pointed out numerous mishaps of forecasting during the pandemic, and it is not our general intent to fix them here. Instead, we start with a basic and yet reasonably effective predictive model for future trends in COVID-19 cases and present a rigorous, quantitative assessment of the added value provided by auxiliary indicators that are derived from data sources that operate outside of traditional public health streams. In particular, we consider five indicators derived from deidentified medical insurance claims, self-reported symptoms from online surveys, and COVID-related Google searches.

To assess this value in as direct terms as possible, we base our study around a very simple basic model: an autoregressive model, in which COVID cases in the near future are predicted using a linear combination of COVID cases in the near past. Forecasting carries a rich literature, offering a wide range of sophisticated techniques (see, e.g., ref. 6 for a review); however, we purposely avoid enhancements such as order selection, correction of outliers/anomalies in the data, and inclusion of regularization or nonlinearities. Similarly, we do not account for other factors that may well aid in forecasting, such as age-specific effects, holiday adjustments, and the effects of public health mandates. All that said, despite its simplicity, the basic autoregressive model that we consider in this paper exhibits competitive performance (see *SI Appendix* for details) with many of the top COVID-19 case forecasters submitted to the US COVID-19 Forecast Hub (7), which is the official source of forecasts used in public communications by the US Centers for Disease Control and Prevention (CDC). The strong performance of the autoregressive model here is in line with the fact that simple, robust models have also consistently been among the best-performing ones for COVID-19 death forecasting (8).

In the companion paper (1), we analyze correlations between various indicators and COVID case rates. These correlations are natural summaries of the contemporaneous association between an indicator and COVID cases, but they fall short of delivering a satisfactory answer to the question that motivates the current article: Is the information contained in an indicator demonstrably useful for the prediction tasks we care about? Note that even lagged correlations cannot deliver a complete answer. Demonstrating utility for prediction is a much higher standard than simply asking about correlations; to be useful in forecast or hotspot models, an indicator must provide relevant information that is not otherwise contained in past values of the case rate series itself [cf. the pioneering work on Granger causality (9, 10), as well as the further references given below]. We assess this directly by inspecting the difference in predictive performance of simple autoregressive models trained with and without access to past values of a particular indicator.

We find that each of the five indicators we consider—three based on COVID-related outpatient visits from medical insurance claims, one on self-reported symptoms from online surveys, and one on Google searches for anosmia or ageusia—provide an overall improvement in accuracy when incorporated into the autorgressive model. This is true both for COVID-19 case forecasting and for hotspot prediction. Further analysis reveals that the gains in accuracy depend on the pandemic’s dynamics at prediction time: The biggest gains in accuracy appear during times in which cases are “flat” or trending “down”; but the indicator based on Google searches offers a notable improvement when cases are trending “up.”

Careful handling of data revisions plays a key role in our analysis. Signals computed from surveillance streams are often subject to latency and/or revision. For example, a signal based on aggregated medical insurance claims may be available after just a few days, but it can then be substantially revised over the next several weeks as additional claims are submitted and/or processed late. Correlations between such a signal and case rates calculated “after the fact” (i.e., computed retrospectively, using the finalized values of this signal) will not deliver an honest answer to the question of whether this signal would have been useful in real time. Instead, we build predictive models using only the data that would have been available as of the prediction date and compare the ensuing predictions in terms of accuracy. The necessity of real-time data for honest forecast evaluations has been recognized in econometrics for a long time (11–21), but it is often overlooked in epidemic forecasting despite its critical importance (22).

Finally, it is worth noting that examining the importance of additional features for prediction is a core question in inferential statistics and econometrics, with work dating back to at least ref. 9. Still today, drawing rigorous inference based on predictions, without (or with lean) assumptions, is an active field of research from both applied and theoretical angles (23–32). Our take in the current work is in line with much of this literature; however, to avoid making any explicit assumptions, we do not attempt to make formal significance statements and, instead, broadly examine the stability of our conclusions with respect to numerous modes of analysis.

## Methods

### Signals and Locations

We consider prediction of future COVID-19 case rates or case hotspots (to be defined precisely shortly). By case rate, we mean the case count per 100,000 people (the standard in epidemiology). We use reported case data aggregated by the Johns Hopkins University Center for Systems Science and Engineering (JHU CSSE) (33), which, like the auxiliary indicators that we use to supplement the basic autoregressive models, is accessible through the COVIDcast API (2).

The indicators we focus on provide information not generally available from standard public health reporting. Among the many auxiliary indicators collected in the API, we study the following five:

• Change Healthcare COVID-like illness (CHNG-CLI): The percentage of outpatient visits that are primarily about COVID-related symptoms, based on deidentified Change Healthcare claims data.
• Change Healthcare COVID (CHNG-COVID): The percentage of outpatient visits with confirmed COVID-19, based on the same claims data.
• COVID Trends and Impact Survey COVID-like illness in the community (CTIS-CLI-in-community): The estimated percentage of the population who know someone in their local community who is sick, based on Delphi’s COVID Trends and Impact Survey, in partnership with Facebook.
• Doctor Visits COVID-like illness (DV-CLI): The same as CHNG-CLI, but computed based on deidentified medical insurance claims from other health systems partners.
• Google search trends for anosmia and ageusia (Google-AA): A measure of Google search volume for queries that relate to anosmia or ageusia (loss of smell or taste), based on Google’s COVID-19 Search Trends dataset.

We choose these indicators because, conceptually speaking, they measure aspects of an individual’s disease progression that would plausibly precede the occurrence of (at worst, co-occur with) the report of a positive COVID-19 test, through standard public health reporting streams.

For more details on the five indicators (including how these are precisely computed from the underlying data streams) we refer to ref. 2 and the companion paper on the COVIDcast API and its signals (1). For CTIS in particular, we refer to the companion paper (34). For the Google COVID-19 Search Trends dataset, see ref. 35; see also refs. 36 and 37 for a justification of the relevance of anosmia or ageusia to COVID infection.

As for geographic resolution, we consider the prediction of COVID-19 case rates and hotspots aggregated at the level of an individual hospital referral region (HRR). HRRs correspond to groups of counties in the United States within the same hospital referral system. *The Dartmouth Atlas of Healthcare 1998* (38) defines these 306 regions based on a number of characteristics. They are contiguous regions such that most of the hospital services for the underlying population are performed by hospitals within the region. Each HRR also contains at least one city where major procedures (cardiovascular or neurological) are performed. The smallest HRR has a population of about 125,000. While some are quite large (such as the one containing Los Angeles, which has more than 10 million people), generally HRRs are much more homogenous in size than the (approximately) 3,200 US counties, and they serve as a nice middle ground in between counties and states.

HRRs, by their definition, would be most relevant for forecasting hospital demand. We have chosen to focus on cases (forecasting and predicting hotspots) at the HRR level because the indicators considered should be more useful in predicting case activity rather than hospital demand, as the former is intuitively more contemporaneous to the events that are measured by the given five indicators. Predicting case rates (and hotspots) at the HRR level is still a reasonable goal in its own right; and moreover, it could be used to feed predicted case information into downstream hospitalization models.

### Vintage Training Data

In this paper, all models are fitted with “vintage” training data. This means that for a given prediction date, say, 28 September 2020, we train models using data that would have been available to us as of 28 September 2020 (imagine that we can “rewind” the clock to 28 September and query the COVIDcast API to get the latest data it would have had available at that point in time). This is possible because of the COVIDcast API’s comprehensive data versioning system [described in more detail in the companion paper (1)]. We also use the evalcast R package (39), which streamlines the process of training arbitrary prediction models over a sequence of prediction dates, by constructing the proper sequence of vintage training datasets.

**Fig. 1.**
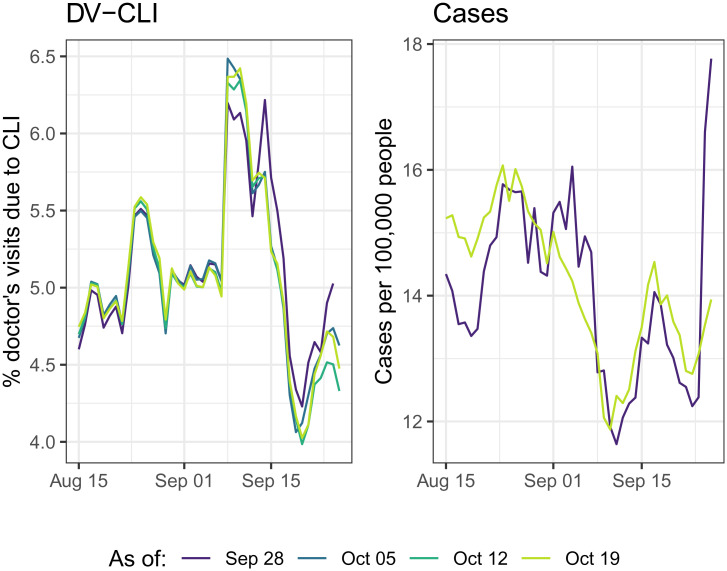
Revision behavior for two indicators in the HRR containing Charlotte, NC. Each colored line corresponds to the data as reported on a particular date (as of dates varying from 28 September through 19 October). (*Left*) The DV-CLI signal, which was regularly revised throughout the period, although the effects fade as we look farther back in time. (*Right*) In contrast, case rates reported by JHU CSSE (smoothed with a 7-d trailing average), which remain “as reported” on 28 September, with a spike toward the end of this period, until a major correction is made on 19 October, which brings this down and affects all prior data as well.

Vintage training data mean different things, in practice, for different signals. The three signals based on medical claims, CHNG-CLI, CHNG-COVID, and DV-CLI, are typically 3 to 5 d latent and subject to a considerable but regular degree of revision or “backfill” after their initial publication date. The survey-based signal, CTIS-CLI-in-community, is 2 d latent and rarely undergoes any revision at all. The target variable itself, reported COVID-19 case rates, is 1 d latent and exhibits frequent, unpredictable revisions after initial publication. Compared to the pattern of revisions in the medical claims signals, which are much more systematic in nature, revisions in case reports can be highly erratic. Big spikes or other anomalies can occur in the data as reporting backlogs are cleared, changes in case definitions are made, etc. Groups like JHU CSSE then work tirelessly to correct such anomalies after first publication (e.g., they will attempt to back distribute a spike when a reporting backlog is cleared, by working with a local authority to ascertain how this should best be done), which can result in very nontrivial revisions. See Fig. 1 for an example.

Finally, our treatment of the Google-AA signal is different from the rest. Because Google’s team did not start publishing this signal until early September 2020, we do not have true vintage data before then; the latency of the signal was always at least 1 wk through 2020. However, unlike the claims-based signals, there is no reason for revisions to occur after initial publication, and furthermore the latency of the signal is not an unavoidable property of the data type, so we simply use finalized signal values, with zero latency, in our analysis.

### Analysis Tasks

To fix notation, let $Y_{\ell ,t}$ denote the 7-d trailing average of COVID-19 case incidence rates in location (HRR) ℓ and at time (day) *t*. To be clear, this is the number of new daily reported cases per 100,000 people, averaged over the 7-d period t−6,…,t. The first task we consider—forecasting—is to predict $Y_{\ell ,t+a}$ for each “ahead” value a=7,…,21. The second task—hotspot prediction—is to predict a binary variable defined in terms of the relative change of $Y_{\ell ,t+a}$ (relative to its value 1 wk prior, $Y_{\ell ,t+a-7}$), again for each a=7,…,21.

Why do we define the response variables via 7-d averaging? The short answer is robustness: Averaging stabilizes the case time series and moderates uninteresting artifacts like day-of-the-week effects in the data. We note that we can also equivalently view this (equivalent up to a constant factor) as predicting the HRR-level case incidence rate summed over some 7-d period in the future and predicting a binary variable derived from this.

In what follows, we cover more details on our two analysis tasks. Table 1 presents a summary.

**Table 1.** ** **Summary of forecasting and hotspot prediction tasks considered in this paper

	Forecasting	Hotspot prediction
Response variable	$Y_{\ell ,t}$ (7-d trailing average of COVID-19 case incidence rates, per location ℓ and time *t*)	$Z_{\ell ,t}=1\left(Y_{\ell ,t}\geq 1.25\cdot Y_{\ell ,t-7}\right)$ (indicator that $Y_{\ell ,t}$ grows by more than 25% relative to the preceding week)
Geographic resolution	HRR	HRR
Forecast period	9 June to 31 December 2020	16 June to 31 December 2020
Model type	Quantile regression	Logistic regression
Evaluation metric	WIS	AUC

#### Dynamic retraining

For each prediction date *t*, we use a 21-d trailing window of data to train our forecast or hotspot prediction models (so, e.g., the trained models will differ from those at prediction date *t* –1). This is done to account for (potential) nonstationarity. For simplicity, the forecasting and hotspot prediction models are always trained on data across all HRRs (i.e., the coefficients in the models do not account for location-specific effects).

#### Prediction period

In our analysis, we let the prediction date *t* run over each day in between early to mid-June and 31 December 2020. The precise start date differs for forecasting and hotspot prediction; for each task it was chosen to be the earliest date at which the data needed to train all models were available, which ends up being (per our setup, with 21 d of training data and lagged values of signals for features, as we detail shortly) 9 June 2020 for forecasting and 16 June 2020 for hotspot prediction. (The bottleneck here is the CTIS-CLI-in-community signal, which does not exist before early April 2020, when the survey was first launched).

#### Forecasting models

Recall that $Y_{\ell ,t}$ denotes the 7-d trailing average of COVID-19 case incidence rates in location ℓ and at time *t*. Separately for each a=7,…,21, to predict $Y_{\ell ,t+a}$ for ahead value *a*, we consider a simple probabilistic forecasting model of the form

[1] $${\text{Quantile}}_{\tau }(Y_{\ell ,t+a}\;|\;Y_{\ell ,s},\;s\leq t)=\alpha^{a,\tau }+\sum_{j=0}^{2}\beta_{j}^{a,\tau }Y_{\ell ,t-7j}.$$

This model uses current case rates, and the case rates 7 and 14 d ago, to predict (the quantiles of) case rates in the future. We consider a total of seven quantile levels (chosen in accordance with the county-level quantile levels suggested by the COVID-19 Forecast Hub),

[2] τ∈{0.025, 0.1, 0.25, 0.5, 0.75, 0.9, 0.975}.

We fit Eq. **1** using quantile regression (40–42) separately for each *τ*, using data from all 306 HRRs and, within each HRR, using the most recent 21 d of training data. This gives us 6,426 training samples for each quantile regression problem.

In addition to this pure autoregressive model, we also consider five probabilistic forecasting models of the form

[3] $$\begin{array}{l} {\text{Quantile}}_{\tau }(Y_{\ell ,t+a}\;|\;Y_{\ell ,s},\;X_{\ell ,s},\;s\leq t)=\\ \;\;\;\;\;\;\;\;\;\;\;\;\alpha^{a,\tau }+\sum_{j=0}^{2}\beta_{j}^{a,\tau }Y_{\ell ,t-7j}+\sum_{j=0}^{2}\gamma_{j}^{a,\tau }X_{\ell ,t-7j}, \end{array}$$

where $X_{\ell ,t}$ denotes any one of the five auxiliary indicators—CHNG-CLI, CHNG-COVID, CTIS-CLI-in-community, DV-CLI, or Google-AA—at location ℓ and time *t*. Note that we apply the same lags (current value, along with the values 7 and 14 d ago) for the auxiliary indicators as we do for the case rates. Training then proceeds just as before: We use the same seven quantile levels in Eq. **2** and fit quantile regression separately for each level *τ*, using data from all 306 HRRs and a trailing window of 21 d of training data.

**Fig. 2.**
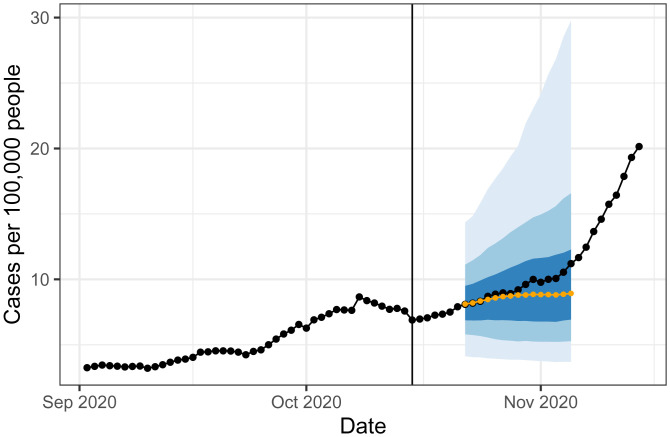
Forecast for the HRR containing New York City from an autoregressive model made on 15 October (vertical line). The fan displays 50, 80, and 95% intervals while the orange curve shows the median forecast. The black curve shows “finalized” data, as reported in May 2021.

At prediction time, to avoid crossing violations (that is, for two levels τ′>τ, the predicted quantile at level *τ* exceeds the predicted quantile at level τ′), we apply a simple post hoc sorting. See Fig. 2 for an example forecast.

#### Hotspot prediction models

Define the binary indicator

$$Z_{\ell ,t}=1\left(Y_{\ell ,t}^{\Delta }\geq 0.25\right),$$

where we use the notation $Y_{\ell ,t}^{\Delta }=(Y_{\ell ,t}-Y_{\ell ,t-7})/(Y_{\ell ,t-7})$. In other words, $Z_{\ell ,t}=1$ if the number of newly reported cases over the past 7 d has increased by at least 25% compared to the preceding week. When this occurs, we say location ℓ is a hotspot at time *t*. Empirically, this rule labels about 27% of location-time pairs as hotspots, during the prediction period (16 June to 31 December 2020).

We treat hotspot prediction as a binary classification problem and use a setup altogether quite similar to the forecasting setup described previously. Separately for each a=7,…,21, to predict $Z_{\ell ,t+a}$, we consider a simple logistic model

[4] $$\text{logit}(\mathbb{P}(Z_{\ell ,t+a}=1\;|\;Y_{\ell ,s},\;s\leq t))=\alpha^{a,\tau }+\sum_{j=0}^{2}\beta_{j}^{a,\tau }Y_{\ell ,t-7j}^{\Delta },$$

where logit(p)=log (p/(1−p)), the log-odds of *p*.

In addition to this pure autoregressive model, we also consider five logistic models of the form

[5] $$\begin{array}{l} \text{logit}(\mathbb{P}(Z_{\ell ,t+a}=1\;|\;Y_{\ell ,s},\;X_{\ell ,s},\;s\leq t))=\\ \;\;\;\;\;\;\;\;\;\;\;\;\;\alpha^{a,\tau }+\sum_{j=0}^{2}\beta_{j}^{a,\tau }Y_{\ell ,t-7j}^{\Delta }+\sum_{j=0}^{2}\gamma_{j}^{a,\tau }X_{\ell ,t-7j}^{\Delta }, \end{array}$$

where we use $X_{\ell ,t}^{\Delta }=(X_{\ell ,t}-X_{\ell ,t-7})/(X_{\ell ,t-7})$, and again $X_{\ell ,t}$ stands for any of the five auxiliary indicators at location ℓ and time *t*. We fit the above models, Eqs. **4** and **5**, using logistic regression, pooling all 306 HRRs and using a 21-d trailing window for the training data.

An important detail is that in hotspot prediction we remove all data from training and evaluation where, on average, fewer than 30 cases (this refers to a count, not a rate) are observed over the prior 7 d. This avoids having to make arbitrary calls for a hotspot (or lack thereof) based on small counts.

### Evaluation Metrics

For forecasting, we evaluate the probabilistic forecasts produced by the quantile models in Eqs. **1** and **3** using weighted interval score (WIS), a quantile-based scoring rule (43). WIS is a proper score, which means that its expectation is minimized by the population quantiles of the target variable. The use of WIS in COVID-19 forecast scoring was proposed by ref. 44; WIS is also the main evaluation metric used in the COVID-19 Forecast Hub. More broadly, the specific form of WIS used here is a standard metric in the forecasting community for evaluating quantile-based probabilistic forecasts, just as mean-squared forecast error is standard for point forecasts.

WIS is typically defined for quantile-based forecasts where the quantile levels are symmetric around 0.5. This is the case for our choice in Eq. **2**. Let *F* be a forecaster composed of predicted quantiles $q_{\tau }$ parameterized by a quantile level *τ*. In the case of symmetric quantile levels, this is equivalent to a collection of central prediction intervals $\left(\ell_{\alpha },u_{\alpha }\right)$, parameterized by an exclusion probability *α*. The WIS of the forecaster *F*, evaluated at the target variable *Y*, is defined by

[6] $$\text{WIS}(F,Y)=\sum_{\alpha }\{\alpha (u_{\alpha }-\ell_{\alpha })+2\cdot \text{dist}(Y,[\ell_{\alpha },u_{\alpha }])\},$$

where dist(a,S) is the distance between a point *a* and set *S* (the smallest distance between *a* and an element of *S*). Note that, corresponding to Eq. **2**, the exclusion probabilities are α∈{0.05, 0.2, 0.5, 1}, resulting in four terms in the above sum. By straightforward algebra, it is not hard to see WIS has an alternative representation in terms of the predicted quantiles themselves:

[7] $$\text{WIS}(F,Y)=2\sum_{\tau }\phi_{\tau }(Y-q_{\tau }),$$

where $\phi_{\tau }(x)=\tau |x|$ for x≥0 and $\phi_{\tau }(x)=(1-\tau )|x|$ for *x* < 0, which is often called the “tilted absolute” loss. While Eq. **7** is more general (it can accommodate asymmetric quantile levels), the first form in Eq. **6** is typically preferred in presentation, as the score nicely decouples into a “sharpness” component (first term in each summand) and an “under/overprediction” component (second term in each summand). But the second form given in Eq. **7** is especially noteworthy in our current study because it reveals WIS to be the same as the quantile regression loss that we use to train our forecasting models.

For hotspot prediction, we evaluate the probabilistic classifiers produced by the logistic models in Eqs. **4** and **5** using the area under the curve (AUC) of their true positive versus false positive rate curve (which is traced out by varying the discrimination threshold).

The primary aggregation scheme that we use in model evaluation and comparisons is to average WIS per forecaster at ahead value *a* over all forecast dates *t* and locations ℓ and, similarly, to compute AUC per classifier at ahead value *a* over all forecast dates *t* and locations ℓ.

### Other Considerations

#### Missing data imputation

Over the prediction period, all auxiliary indicators are available (in the proper vintage sense) for all locations and prediction times, except for the Google-AA signal, which is observed only for an average of 105 (of 306) HRRs. Such missingness occurs because the COVID-19 search trends data are constructed using differential privacy methods (45), and a missing signal value means that the level of noise added in the differential privacy mechanism is high compared to the underlying search count. In other words, values of the Google-AA signal are clearly not missing at random. It seems most appropriate to impute missing values by zero, and this is what we do in our analysis.

#### Backfill and nowcasting

As described previously, the auxiliary indicators defined in terms of medical claims (CHNG-CLI, CHNG-COVID, and DV-CLI) undergo a significant and systematic pattern of revision, or backfill, after their initial publication. Given their somewhat statistically regular backfill profiles, it would be reasonable to attempt to estimate their finalized values based on vintage data—a problem we refer to as nowcasting—as a preprocessing step before using them as features in the models in Eqs. **3** and **5**. Nowcasting is itself a highly nontrivial modeling problem, and we do not attempt it in this paper (it is a topic of ongoing work in our research group), but we note that nowcasting would likely improve the performance of the models involving claims-based signals in particular.

#### Spatial heterogeneity

Some signals have a significant amount of spatial heterogeneity, by which we mean their values across different geographic locations are not comparable. This is the case for the Google-AA signal (due to the way in which the Google search trends time series is self-normalized) (35) and the claims-based signals (due to market-share differences and/or differences in health-seeking behavior). Such spatial heterogeneity likely hurts the performance of the predictive models that rely on these signals, because we train the models on data pooled over all locations. In the current paper, we do not attempt to address this issue, and we simply note that location-specific effects (or preprocessing to remove spatial bias) would likely improve the performance of the models involving Google-AA and the claims-based indicators.

## Results

Here, and in what follows, we will use “AR” to refer to the pure autoregressive model both in forecasting (Eq. **1**) and in hotspot prediction (Eq. **4**) (the reference to the prediction task should always be clear from the context). We will also use the name of an auxiliary indicator—namely “CHNG-CLI,” “CHNG-COVID,” “CTIS-CLI-in-community,” “DV-CLI,” or “Google-AA”—interchangeably with the model in forecasting (Eq. **3**) or hotspot prediction (Eq. **5**) that uses this same indicator as a feature (the meaning should be clear from the context). So, for example, the CHNG-CLI model in forecasting is the one in Eq. **3** that sets $X_{\ell ,t}$ to be the value of the CHNG-CLI indicator at location ℓ and time *t*. Finally, we use the term “indicator model” to refer to any one of the 10 models of the form Eq. **3** or **5** (5 such models for each of the forecasting and hotspot prediction tasks).

Below is a summary of the high-level conclusions:

• Stratifying predictions by the ahead value (a=7,…,21) and aggregating results over the prediction period (early June through end of December 2020), we find that each of the indicator models generally gives a boost in predictive accuracy over the AR model, in both the forecasting and hotspot prediction tasks. The gains in accuracy generally attenuate as the ahead value grows.
• In the same aggregate view, CHNG-COVID and DV-CLI offer the biggest gains in both forecasting and hotspot prediction. CHNG-CLI is inconsistent: It provides a large gain in hotspot prediction, but little gain in forecasting (it seems to be hurt by a notable lack of robustness, due to backfill). CTIS-CLI-in-community and Google-AA each provide decent gains in forecasting and hotspot prediction. The former’s performance in forecasting is notable in that it clearly improves on AR even at the largest ahead values.
• In a more detailed analysis of forecasting performance, we find that the indicator models tend to be better than AR when case rates are flat or decreasing (most notable in CHNG-COVID and CTIS-CLI-in-community), but can be worse than AR when case rates are increasing (this is most notable in CHNG-CLI and DV-CLI). More rarely does an indicator model tend to beat AR when case rates are increasing, but there appears to be evidence of this for the Google-AA model.
• In this same analysis, when an indicator model performs better than AR in a decreasing period, this tends to co-occur with instances in which the indicator “leads” case rates (meaning, roughly, on a short timescale in a given location, its behavior mimics that of future case rates). On the other hand, if an indicator model does better in periods of increase, or worse in periods of increase or decrease, its performance is not as related to leadingness.

Finally, to quantify the importance of training and making predictions using proper vintage data, we ran a parallel set of forecasting and hotspot prediction experiments using finalized data. The results, given in *SI Appendix*, show that training and making predictions on finalized data can result in overly optimistic estimates of true test-time performance (up to 10% better in terms of average WIS or AUC). Furthermore, since indicators can have greatly different backfill profiles, the use of finalized data in retrospective evaluations changes the relative ranking of models. For example, CHNG-CLI and DV-CLI, when trained on finalized data, perform very similarly in forecasting. This makes sense since they are both claims-based indicators that are supposedly measuring the same thing. However, DV-CLI outperforms CHNG-CLI on vintage data, reflecting that it has a less severe backfill profile.

*SI Appendix* provides a number of other additional analyses; for example, we examine two assumption-lean methods for assessing the statistical significance of our main results.

Code to reproduce all results can be found in ref. 2.

### Aggregate Results by Ahead Value

**Fig. 3.**
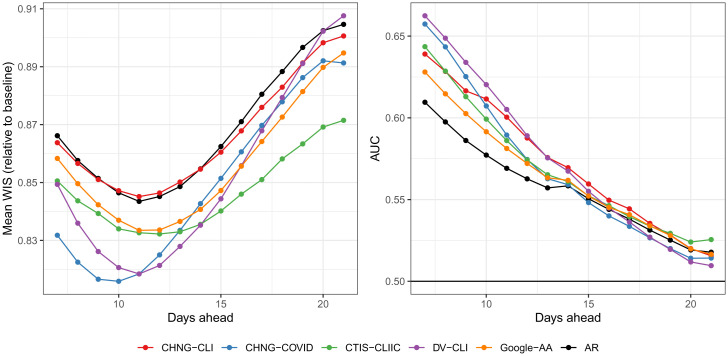
Main results for both tasks. (*Left*) Average WIS for each forecast model, over all forecast dates and all HRRs, divided by the average WIS achieved by a baseline model (a probabilistic version of the flat-line forecaster). (*Right*) Area under the curve for each hotspot prediction model, calculated over all prediction dates and all HRRs. Here and in all figures we abbreviate CTIS-CLI-in-community by CTIS-CLIIC.

Fig. 3, *Left* displays evaluation results for forecasting, stratified by ahead value and averaged over all HRRs and forecast dates. Shown is the average WIS for each forecast model divided by that from a baseline model. The baseline model is a flat-line forecaster that forms its median forecast by using the most recent value $Y_{\ell ,t}$ for all aheads $Y_{\ell ,t+a}$, with predicted quantiles defined by the empirical distribution of the residuals from this median forecast over recent history. This is the same baseline model as in the COVID-19 Forecast Hub. Here, we use the baseline model to scale mean WIS to put it on an interpretable, unitless scale. In Fig. 3, *Left* we can see that all curves are below 1, which means (smaller WIS is better) that all of the models, including AR, outperform the baseline on average over the forecasting period. On the other hand, the models deliver at best an improvement of about 20% in average WIS over the baseline model, with this gap narrowing to about 10% at the largest ahead values, illustrating the difficulty of the forecasting problem.

We can also see from Fig. 3, *Left* that CHNG-COVID and DV-CLI offer the biggest gains over AR at small ahead values, followed by CTIS-CLI-in-community and Google-AA, with the former providing the biggest gains at large ahead values. The CHNG-CLI model performs basically the same as AR. This is likely due to the fact that CHNG-CLI suffers from volatility due to backfill. The evidence for this explanation is twofold: 1) The CHNG-CLI model benefits from a more robust method of aggregating WIS (geometric mean; shown in *SI Appendix*), and 2) when we train and make predictions on finalized data, it handily beats AR, on par with the best-performing models (also shown in *SI Appendix*).

Fig. 3, *Right* displays the results for hotspot prediction, again stratified by ahead value and averaged over all HRRs and prediction dates. We can see many similarities to the forecasting results (note that larger AUC is better). For example, CHNG-COVID and DV-CLI offer the biggest improvement over AR, and all models, including AR, degrade in performance toward the baseline (in this context, a classifier based on random guessing, which achieves an AUC of 0.5) as the ahead values grow, illustrating the difficulty of the hotspot prediction problem. A clear difference, however, is that the CHNG-CLI model performs quite well in hotspot prediction, close to the best-performing indicator models for many of the ahead values. This may be because volatility in the CHNG-CLI indicator plays less of a role in the associated logistic model’s predicted probabilities (in general, a sigmoid function can absorb a lot of the variability in its input).

### Implicit Regularization Hypothesis

One might ask whether the benefits observed in forecasting and hotspot prediction have anything to do with the actual auxiliary indicators themselves. A plausible alternative explanation is that the indicators are just providing implicit regularization on top of the basic AR model, in the same way any noise variable might, if we were to use it to create lagged features in Eqs. **3** and **5**.

To test this hypothesis, we reran all of the prediction experiments but with $X_{\ell ,t}$ in each indicator model replaced by suitable random noise (bootstrap samples from a signal’s history). The results, shown and explained more precisely in *SI Appendix*, are vastly different (i.e., worse) than the original set of results. In both forecasting and hotspot prediction, the “fake” indicator models offer essentially no improvement over the pure AR model, which—informally speaking—strongly rejects the implicit regularization hypothesis.

On the topic of regularization, it is also worth noting that the use of $\ell_{1}$ regularization (tuned using cross-validation) in fitting any of the models in Eqs. **1**, **3**, **4**, and **5** did not generally improve their performance (experiments not shown). This is likely due to the fact that the number of training samples is large compared to the number of features (6,426 training samples and only three to six features).

### Evaluation in Up, Down, and Flat Periods

The course of the pandemic has played out quite differently across space and time. Aggregating case rates nationally shows three pronounced waves, but the behavior is more nuanced at the HRR level. Fig. 2 is a single example of a forecast in a period of relatively flat case trends, as New York City enters what would become its second wave. The AR forecaster’s 50% prediction interval contains this upswing, but its forecasted median is clearly below the finalized case data. Unfortunately, this behavior is fairly typical of all forecasters: During upswings, the forecasted median tends to fall below the target, while the reverse is true during downswings.

**Fig. 4.**
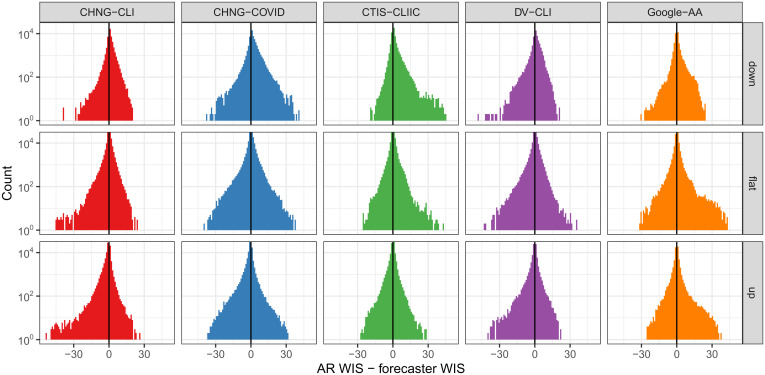
Histogram of the difference in WIS for the AR model and that for each indicator model, stratified by up, down, or flat period, measured in terms of case trends. Note that larger differences here are better for each indicator model. The *y* axis is on the log scale to emphasize tail behavior.

Fig. 4 shows histograms of the differences in WIS of the AR model and each indicator model, where we stratify these differences by whether the target occurs during a period of increasing case rates (up), decreasing case rates (down), or flat case rates (flat). To define the increasing period, we use the same definition we used for the hotspot task in Table 1. Therefore, all hotspots are labeled up, while all nonhotspots are either flat or down. For the down scenario, we simply use the opposite of the hotspot definition: $Y_{\ell ,t}$ decreases by more than 20% relative to the preceding week.

While the performance of all models, including AR, generally degrades in up periods, different models exhibit different and interesting patterns. CHNG-CLI, CHNG-COVID, Google-AA, and especially CTIS-CLI-in-community have large right tails (showing improvements over AR) during the down periods. Google-AA and CTIS-CLI-in-community have large right tails during the flat periods. CHNG-CLI and DV-CLI have large left tails (poor forecasts relative to AR) in flat and up periods. Google-AA is the only model that outperforms the AR model, on average, in up periods. Overall, the indicators seem to help more during flat or down periods than during up periods, with the exception of Google-AA.

*SI Appendix* pursues this analysis further. For example, we examine classification accuracy and log-likelihood for the hotspot task and find a similar phenomenon: The indicators considerably improve accuracy or log-likelihood during flat or down periods, with more mixed behavior during up periods when CHNG-CLI, CHNG-COVID, and DV-CLI, in particular, lead to decreased performance.

### Effects of Leading or Lagging Behavior

As described in *Methods*, each of the indicators we examine could be said to measure aspects of disease progression that would precede a positive test. That is, we imagine that these signals should “lead” cases. It is entirely reasonable to imagine that, prior to an increase of confirmed COVID-19 tests reported by a public health authority in a particular location, we would see an increase in medical insurance claims for COVID-related outpatient visits. However, it may well be the case that such behavior is different during different periods. In fact, we find empirically that the “leadingness” of an indicator (degree to which it leads case activity) tends to be more pronounced in down or flat periods than in up periods, a plausible explanation for the decreased performance in up periods noted above.

**Fig. 5.**
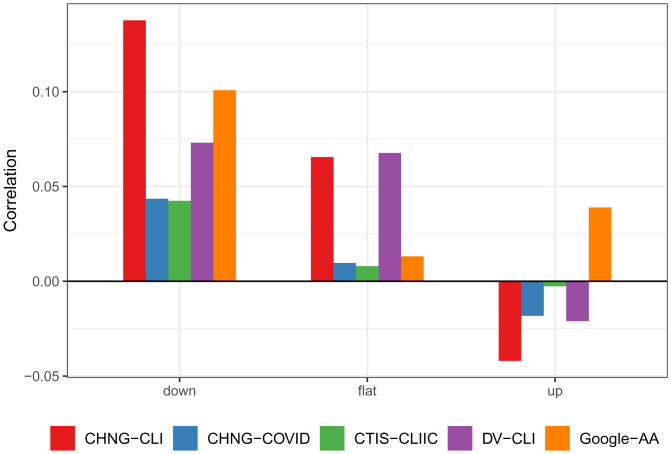
Correlation of the difference in WIS with the leadingness of the indicator at the target date, stratified by up, down, or flat period.

In *SI Appendix*, we define a quantitative score to measure the leadingness of an indicator, at any time *t* and any location ℓ, based on cross-correlations to case rates over a short time window around *t*. The higher this score is, the greater it leads case activity. This analysis is closely related to Granger causality (10) and draws on a large body of prior work that measures leadingness in economic time series (9, 46–57). Fig. 5 displays correlations between the leadingness score of an indicator and the WIS difference (AR model minus an indicator model), stratified by whether the target is classified as up, down, or flat. One would naturally expect that the WIS difference would be positively correlated with leadingness. Somewhat surprisingly, this relationship turns out to be strongest in down periods and weakest in up periods.

In fact, it is very nearly the case that for each indicator, the strength of correlations only decreases as we move from down to flat to up periods. In *SI Appendix*, we extend this analysis by studying analogous “laggingness” scores, but we do not find as clear patterns.

## Discussion

Can auxiliary indicators improve COVID-19 forecasting and hotspot prediction models? Our answer, based on analyzing five auxiliary indicators from the COVIDcast API (defined using data from medical insurance claims, internet-based surveys, and internet search trends) is undoubtedly “yes.” However, there are levels of nuance to such an answer that must be explained. None of the indicators that we have investigated are transformative, rendering the prediction problem easy when it was once hard (in the absence of auxiliary information). Rather, the gains in accuracy from the indicator models (over an autoregressive model based only on past case rates) appear to be nontrivial and consistent across modes of analysis, but modest. In forecasting, the indicator models are found to be most useful in periods in which case rates are flat or trending down, rather than in periods in which case rates are trending up (as one might hope to see is the benefit being provided by a hypothetical “leading indicator”).

As described previously, it is likely that we could improve the indicator models by using location-specific effects, as well as using nowcasting techniques to estimate finalized indicator values before we use them as features (to account for backfill in the claims-based signals in particular). Beyond this, it is certainly possible that more sophisticated models for forecasting or hotspot prediction would lead to different results and possibly even different insights. Natural directions to explore include using multiple indicators in a single model, allowing for interaction terms, and leveraging HRR demographics or mobility patterns. That said, we are doubtful that more sophisticated modeling techniques would change the “topline” conclusion—that auxiliary indicators can provide clear but modest gains in forecasting and hotspot prediction.

However, rigorously vetting the details for more sophisticated models, as well as the generalizability of our findings to different geographic resolutions, both remain important directions for future study. For example, in *SI Appendix* we show that for forecasting at the state level, the benefits of including indicators in the AR model are generally less clear (compared to those observed at the HRR level). A plausible explanation is that at the state level, where the signal-to-noise ratio (SNR) is higher, AR performs better overall and represents a higher standard (when asking whether it can be improved upon using the indicators). At the HRR level, where the SNR is lower, including the indicators as additional linear features in the AR model probably delivers a kind of variance reduction (just like averaging independent terms would) that contributes to improved accuracy. But as the SNR increases, this variance reduction becomes less important, and perhaps we must use more sophisticated modeling techniques to extract comparable value from the indicators.

We reiterate the importance of using vintage data for rigorous backtesting. Data sources that are relevant to public health surveillance are often subject to revision, sometimes regularly (as in medical claims data) and sometimes unpredictably (such as COVID-19 case reports). When analyzing models that are designed to predict future events, if we train these models and make predictions using finalized data, then we are missing a big part of the story. Not only will our sense of accuracy be unrealistic, but also certain models may degrade by a greater or lesser extent when they are forced to reckon with vintage data, so backtesting on finalized data may lead us to make modeling decisions that are suboptimal for true test-time performance.

In this paper, we have chosen to consider only very simple forecasting models, while devoting most of our effort to accounting for as much of the complexity of the underlying data and evaluation as possible. In fact, our paper is not only about providing rigorous answers to questions about model comparisons in COVID-19 forecasting and hotspot prediction, but also about demonstrating how one might go about answering such questions in general. We hope that others will leverage our work, and build on it, to guide advances on the frontier of predictive modeling for epidemics and pandemics.

## Supplementary Material

Appendix 01 (PDF)

## Data Availability

Code for reproducibility, R script files, and small datasets have been deposited in Zenodo (58). Large intermediate datasets, archived.rds (R objects) files containing all evaluations for forecasting, and hotspots using vintage and finalized data (can be used to produce the graphics and conclusions in the paper without rerunning the entire pipeline) are available in the University of British Columbia Scholars Portal Dataverse (59).
