# Supplementary material for: Can auxiliary indicators improve COVID-19 forecasting and hotspot prediction?
Source: Proc Natl Acad Sci U S A. 2021 Dec 13;118(51):e2111453118. doi: 10.1073/pnas.2111453118 (PMC8713796; doi:10.1073/pnas.2111453118)
Supplement: Supplementary File [file pnas.2111453118.sapp.pdf]

PNAS
www.pnas.org

Supplementary Information for

Can Auxiliary Indicators Improve COVID-19 Forecasting and Hotspot Prediction?

Daniel J. McDonald, Jacob Bien, Alden Green, Addison J. Hu, Nat DeFries, Sangwon Hyun, Natalia L. Oliveira, James Sharpnack, Jingjing Tang, Robert Tibshirani, Valerie Ventura, Larry Wasserman, and Ryan J. Tibshirani

Daniel J. McDonald.
E-mail: daniel@stat.ubc.ca

This PDF file includes:

Supplementary text
Figs. S1 to S21 (not allowed for Brief Reports)
Table S1 (not allowed for Brief Reports)
Legends for Dataset S1 to S2
SI References

Other supplementary materials for this manuscript include the following:

Datasets S1 to S2

Supporting Information Text

1. Finalized Versus Vintage Data

The goal of this section is to quantify the effect of not properly accounting for the question of "what was known when" in performing retrospective evaluations of forecasters. Figures S1 and S2 show what Figures 3 and 4 in the main paper would have looked like if we had simply trained all models using the finalized data rather than using vintage data. This comparison can be seen more straightforwardly in Figures S3 and S4, which show the ratio in performance between the vintage and finalized versions. When methods are given the finalized version of the data rather than the version available at the time that the forecast would have been made, all methods appear (misleadingly) to have better performance than they would have had if run prospectively. For example, for forecasting case rates 7-days ahead, the WIS of all methods is at least 8% larger than what would have been achieved using finalized data. This effect diminishes as the forecasting horizon increases, reflecting the fact that longer-horizon forecasters rely less heavily on recent data than very short-horizon forecasters. Crucially, some methods are "helped" more than others by the less scrupulous retrospective evaluation, underscoring the difficulty of avoiding misleading conclusions when performing retrospective evaluations of forecasters.

CHNG-CLI (and, to a lesser extent, the other claims-based signals) is the most affected by this distinction, reflecting the latency in claims-based reporting. This highlights the importance of efforts to provide "nowcasts" for claims signals (which corresponds to a 0-ahead forecast of what the claims signal's value will be once all data has been collected). Looking at the CHNG-CLI and DV-CLI curves in Figure S1, we can see that they perform very similarly when trained on the finalized data. This is reassuring because they are, in principle, measuring the same thing (namely, the percentage of outpatient visits that are primarily about COVID-related symptoms), but based on data from different providers. The substantial difference in their curves in Figure 3 of the main paper must, therefore, reflect their having very different backfill profiles.

While using finalized rather than vintage data affects DV-CLI the least for forecasting, it is one of the most affected methods for the hotspot problem. This is a reminder that the forecasting and hotspot problems are fundamentally different problems. For example, the hotspot problem does not measure the ability to distinguish between flat and downward trends.

Even the AR model is affected by this distinction, reflecting the fact that the case rates themselves (i.e., the response values) are also subject to revision. The forecasters based on indicators are thus affected both by revisions to the indicators and by revisions to the case rates. And in the case of the Google-AA model, in which we only used finalized values for the Google-AA indicator, the difference in performance can be wholly attributed to revisions of case rates.

2. Robust Aggregation

In this section, we consider using the geometric mean instead of the usual (arithmetic) mean when aggregating the weighted interval score (WIS) across location-time pairs. Aside from the geometric mean being generally more robust to large values, there are two reasons why using it may be desirable.

1. WIS is right-skewed, being bounded below by zero and having occasional very large values. Figure S5 illustrates that the densities appear roughly log-Gaussian. The geometric mean is a natural choice in such a context since it can be viewed as a measure of centrality on the log scale (it is the exponential of the arithmetic mean of log values).

2. In the main paper, we report the ratio of the mean WIS of a forecaster to the mean WIS of the baseline forecaster. Another choice could be to take the mean of the ratio of WIS values for the two methods. This latter choice would penalize a method less for doing poorly where the baseline forecaster also does poorly.* Using instead the geometric mean makes the order of aggregation and scaling immaterial since the ratio of geometric means is the same as the geometric mean of ratios.

Figure S6 uses the geometric mean for aggregation. Comparing this with Figure 3 in the main paper, we see that the main conclusions are largely unchanged; however, CHNG-CLI now appears better than AR. This behavior would be expected if CHNG-CLI's poor performance is attributable to a relatively small number of large errors (as opposed to a large number of moderate errors). Indeed, Figure 5 of the main paper further corroborates this, in which we see the heaviest left tails occur for CHNG-CLI.

3. Comparing COVID-19 Forecast Hub Models

Since July of 2020, modelers have been submitting real-time forecasts of COVID-19 case incidence to the COVID-19 Forecast Hub (1). This (along with forecasts of hospitalizations and deaths collected in the same Hub) serves as the source of the CDC's official communications on COVID forecasting.

Our goal in this section is to compare the AR model and indicator models to those in the Hub, in terms of forecast errors aggregated over the same forecast tasks, to give a clear sense for how robust and effective the models we choose to investigate in the paper are relative to those in common operational use. This was prompted by a question from an anonymous reviewer of this paper, who asked why we chose to build our analysis of indicator utility around the AR model in the first place, and why

*In a sense, this is implicitly estimating a nonparametric space-time effect for forecaster error, and assuming that has a shared, multiplicative contribution to forecaster errors. That is, if one imagines that a forecaster's WIS is composed of multiplicative space-time effects $S_{\ell,t}$ shared across all forecasters, $\mathrm{WIS}(F_{\ell,t,f}, Y_{\ell,t}) = S_{\ell,t} E_{f,t}$ with $E_{f,t}$ a forecaster-specific error, then taking the ratio of individual WIS values cancels these space-time effects.

we did not build it around others (say, the SIR model or more complex mechanistic models of disease transmission) that have occupied more of the spotlight over the course of the pandemic. The analysis presented here corroborates the claim that, while simple, the AR model, properly trained—using a quantile loss to directly estimate multiple conditional quantiles, a trailing training window of 21 days, pooling across all locations jointly, and fitting to case rates rather than counts (as we do in all our models in the main paper)—can be robust and effective, performing competitively to the top models submitted to the COVID-19 Forecast Hub, including the Hub's ensemble model.

The closest forecast target in the Hub to that used in the main paper is state-level case incidence over an epiweek—defined by the sum of new case counts reported between a Sunday and the following Saturday (inclusive). Our forecast target, recall, is a 7-day trailing average of COVID-19 case incidence rates at the HRR level, which is different in three regards:

1. temporal resolution (daily versus weekly);

2. geographic resolution (HRRs versus states);

3. scale (rates versus counts).

While the first and third of these differences could be easily addressed post hoc—meaning, we can take always take our model's output and multiply it by 7 in order to track the incidence over any given trailing week, and rescale it per location by population to bring it to the count scale—the second difference is not easy to adjust post hoc due to nonlinearity of the quantiles (a quantile of a linear combination of random variables is not simply the linear combination of their quantiles, but rather, depends intricately on the correlations between the random variables).

Therefore, to make the comparison to models in the Hub as direct as possible, we retrained our models over the same forecast period as in the main paper, and with the same general setup entirely, except at the state rather than HRR level. We then rescaled them post hoc to account for the different temporal resolution and the rate-versus-count scaling (first and third points in the above list). The results are given in Figure S7. The evaluation was carried out exactly as in the main paper, and the figure displays both mean WIS and geometric mean WIS, as a function of ahead, relative to the baseline model. Furthermore, to account for missingness (not all teams submitted forecasts to the Hub for all locations and ahead values for the entire period), we first dropped any forecaster than submitted for less than 6 weeks, and then restricted the aggregation metrics (mean or geometric mean) to errors from commonly available forecast tasks.

By either metric, mean or geometric mean WIS relative to baseline, we can see in Figure S7 that the AR model examined in this paper is competitive with top models in the Hub, even outperforming the Hub ensemble model for smaller ahead values. The same general conclusion can be drawn for the indicator-assisted models as well. However, interestingly, a close inspection reveals that the AR model here is for the most part in the "middle of the pack" when compared to the indicator models, and only the Google-AA model offers clear improvement over AR for all aheads. This is likely due to the fact that at the state level, the signal-to-noise ratio (SNR) is generally higher, and AR model provides a higher standard (on which to expect improvement using an auxiliary indicator), since it is able to extract a clearer signal from past lags of case rates. At the HRR level, with lower SNR, using indicators as simple additional linear features in the AR model probably leads to a variance reduction that is enough to boost accuracy, but at the state level, perhaps more sophisticated modeling techniques are needed to extract comparable value from some of the indicators.

4. Statistical Significance

In the introduction of the main manuscript, we gave some reasons that we avoid making formal statements about statistical significance, preferring instead to examine the stability of our results in different contexts. There are strong reasons to avoid model-based significance tests because the necessary assumptions about stationarity, independence, and the model being true (or at least approximately true) are certainly violated. With those caveats in mind, we undertake two relatively assumption-lean investigations in this section. The first is a sign test for whether the difference between the AR model's relative WIS and each other model's relative WIS is centered at zero. (Relative WIS here means scaled by the WIS of the baseline model.) To mitigate the dependence across time (which intuitively seems to matter more than that across space), we computed these tests in a stratified way, where for each forecast date we run a sign test on the scaled errors between two models over all 306 HRRs. The results are plotted as histograms in Figure S8. For this figure, we use the total relative WIS over all aheads, but the histograms are largely similar for individual target horizons. If there were no difference, we would expect to see a uniform distribution. However, for each indicator model, we see many more small p-values than would be expected if the null hypothesis (that the AR model is better) were true.

Another relatively assumption-lean method of testing for differences in forecast accuracy is the Diebold-Mariano (DM) test (2–4). Essentially, the differences between forecast errors are assumed to have a constant mean and a covariance that depends on time. Under these conditions, the asymptotic distribution for the standardized mean of the differences is limiting normal provided that a heteroskedasticity and autocorrelation robust estimate of the variance is used. Using the error as WIS across all HRRs and horizons (7 to 21 days ahead), we perform the DM test using both the mean relative to the baseline (as reported in the manuscript) and the geometric mean relative to the baseline as described above. The first two rows of Table S1 displays p-values for the test that each indicator model is no better than the AR model. In only a few instances—geometric mean and mean for the CHNG-CLI model, and mean for the CHNG-COVID model—do the p-values exceed conventional statistical significance thresholds.

5. Bootstrap Results

As explained in the *Implicit regularization hypothesis* section of the main paper, a (somewhat cynical) hypothesis for why we see benefits in forecasting and hotspot prediction is that the indicators are not actually providing useful information but they are instead acting as a sort of "implicit regularization," leading to shrinkage on the autoregressive coefficients and therefore to less volatile predictions. To investigate this hypothesis, we consider fitting "noise features" that in truth have zero relationship to the response. Recall (from the main paper) that at each forecast date, we train a model on 6,426 location-time pairs. Each indicator model uses 6 features, corresponding to the 3 autoregressive terms and the 3 lagged indicator values. To form noise indicator features, we replace their values with those from a randomly chosen time-space pair (while keeping the autoregressive features fixed). In particular, at each location ℓ and time t, for the forecasting task we replace the triplet $(X_{\ell,t}, X_{\ell,t-7}, X_{\ell,t-14})$ in Eq. (3) of the main paper with the triplet $(X_{\ell^*,t^*}, X_{\ell^*,t^*-7}, X_{\ell^*,t^*-14})$, where (ℓ^*, t^*) is a location-time pair sampled with replacement from the 6,426 total location-time pairs. Likewise in the hotspot prediction task, we replace the triplet $(X^{\triangle}_{\ell,t}, X^{\triangle}_{\ell,t-7}, X^{\triangle}_{\ell,t-14})$ in Eq. (5) of the main paper with $(X^{\triangle}_{\ell^*,t^*}, X^{\triangle}_{\ell^*,t^*-7}, X^{\triangle}_{\ell^*,t^*-14})$. Figures S9–S11 show the results. No method exhibits a noticeable performance gain over the AR method, leading us to dismiss the implicit regularization hypothesis.

6. Upswings and Downswings

In this section, we provide extra details about the upswing/downswing analysis described in the main manuscript in the section titled *Evaluation in Up, Down, and Flat Periods.* Figure S12 shows the overall results, examining the average difference $\mathrm{WIS(AR)} - \mathrm{WIS}(F)$ for each forecaster F, in each in each period. Figure S13 shows the same information for the hotspot task. On average, during downswings and flat periods, the indicator models have lower classification error and higher log likelihood than the AR model. For hotspots, both Google-AA and CTIS-CLIIC perform better than the AR model during upswings, in contrast to the forecasting task, where only Google-AA improves. In a related analysis, Figure S14 shows histograms of the Spearman correlation (Spearman's ρ, a rank-based measure of association) between the ratio $\mathrm{WIS}(F)/\mathrm{WIS(AR)}$ and the magnitude of the swing. Again we see that case rate increases are positively related to diminished performance of the indicator models.

One hypothesis for diminished relative performance during upswings is that the AR model tends to overpredict downswings and underpredict upswings. Adding indicators appears to help avoid this behavior on the downswing but not as much on upswings. Figure S15 shows the correlation between between $\mathrm{WIS(AR)} - \mathrm{WIS}(F)$ and the difference of their median forecasts. During downswings, this correlation is large, implying that improved relative performance of F is related to making lower forecasts than the AR model. The opposite is true during upswings. This is largely to be expected. However, the relationship attenuates in flat periods and during upswings. That is, when performance is better in those cases, it may be due to other factors than simply making predictions in the correct direction, for example, narrower confidence intervals.

It is important to note that, even though some indicators—notably CHNG-COVID and CHNG-CLI—underperform relative to the AR model during upswings, all models dramatically outperform the baseline in such periods. Furthermore, Figure S16 shows the performance of all forecasters relative to the baseline model. All forecasters suffer relative to the baseline during down periods, but the AR is the worst. In contrast, all models beat the baseline during up periods, even CHNG-COVID and CHNG-CLI, though not by quite as much as the AR does.

7. Leadingness and Laggingness

In the section titled *Effects of Leading or Lagging Behavior* of the main text, we discuss the extent to which the indicators are leading or lagging case rates during different periods. To define the amount of leadingness or laggingness at location ℓ, we use the cross correlation function (CCF) between the two time series. The $\mathrm{CCF}_\ell(a)$ of an indicator series X_ℓ and case rate series Y_ℓ is defined as their Pearson correlation where X_ℓ has been aligned with the values of Y_ℓ that occurred a days earlier. Thus, for any $a > 0$, $\mathrm{CCF}_\ell(a) > 0$ indicates that $Y_{\ell,t}$ is moving together with $X_{\ell,t+a}$. In this case we say that X_ℓ is lagging Y_ℓ. For $a < 0$, $\mathrm{CCF}_\ell(a) > 0$ means that $Y_{\ell,t}$ is positively correlated with $X_{\ell,t-a}$, so we say that X_ℓ leads Y_ℓ.

Figure S17 shows the standardized signals for the HRR containing Charlotte, North Carolina, from August 1, 2020 until the end of September. These are the same signals shown in Figure 1 in the manuscript, but using finalized data. To define "leadingness" we compute $\mathrm{CCF}_\ell(a)$ (as implemented with the R function `ccf()`) for each $a \in \{-15, \ldots, 15\}$ using the 56 days leading up to the target date. This is the same amount of data used to train the forecasters: 21 days of training data, 21 days to get the response at $a = 21$, and 14 days for the longest lagged value. The orange dashed horizontal line represents the 95% significance threshold for correlations based on 56 observations. Any correlations larger in magnitude than this value are considered statistically significant under the null hypothesis of no relationship. We define leadingness to be the sum of the significant correlations that are leading (those above the dashed line with $a < 0$) while laggingness is the same but for $a > 0$. In the figure, there are three significant correlations on the "leading" side (at $a = -5, -4, -3$), so leadingness will be the sum of those values while laggingness is 0: on September 28 in Charlotte, DV-CLI is leading cases leading but not lagging.

Figure S18 shows the correlation between laggingness and the difference in forecaster WIS and AR WIS. Unlike leadingness (Figure 5 in the manuscript) there is no obvious relationship that holds consistently across indicators. This is encouraging as laggingness should not aid forecasting performance. On the other hand, if an indicator is more lagging than it is leading, this may suggest diminished performance. Figure S19 shows the correlation of the difference in leadingness and laggingness with the difference in WIS. The pattern here is largely similar to the pattern in leadingness described in the manuscript: the

relationship is strongest in down periods and weakest in up periods with the strength diminishing as we move from down to flat to up for all indicators.

In calculating the CCF and the associated leadingness and laggingness scores, we have used the finalized data, and we look at the behavior at the target date of the forecast. That is we are using the same data to evaluate predictive accuracy as to determine leadingness and laggingness. It should be noted that the leadingness of the indicator at the time the model is trained may also be important. Thus, we could calculate separate leadingness and laggingness scores for the trained model and for the evaluation data and examine their combination in some way. However, we do not pursue this further.

8. Disaggregation Over Time and Space

Following the suggestion of an anonymous reviewer, we investigate two other disaggregated versions of the main forecasting result shown in Figure 3 on the manuscript. Below we use the term "error" to refer to the WIS summed over all ahead values. The first (Figure S20) displays the cumulative error, up through any point in time, of each forecaster divided by the cumulative error of the baseline. This perspective should illustrate how the models perform over time, drawing attention to any nonstationary behavior. During the initial increase in cases in July 2020, CTIS-CLIIC and Google-AA gain quite a bit acurracy compared to the AR model. All the indicators do much better than the AR model during the following downturn (the ebb of the second wave). The AR model actually improves over the indicators in October 2020, before losing a bit in late November.

Figure S21 examines the spatial behavior of errors over the entire period of the indicator models relative to the AR. For ease of comparison, we show the percent improvement in each HRR. Negative numbers (blue) mean that the indicator helped while positives (red) mean that the indicator hurt forecasting performance. The clear pattern is that in most HRRs, the indicators improved performance, though usually by small amounts (2.5%–10%). In some isolated HRRs, performance was markedly worse, though there does not appear to be any particular pattern to these locations. Interestingly, the geographic patterns of improvement differ quite a lot in between the indicators. This suggests that a forecasting model that carefully combines all of indicators could be a considerable improvement.

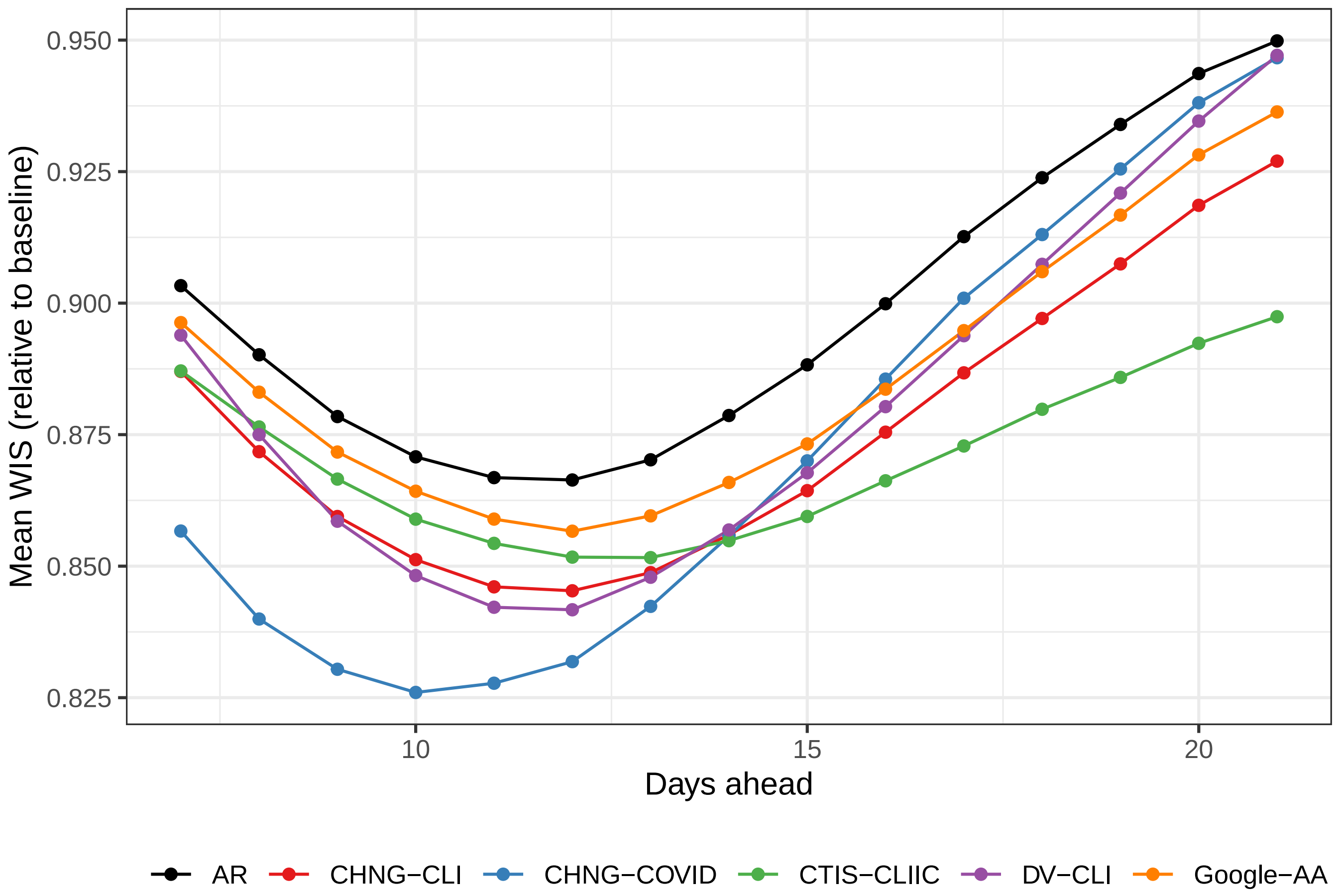

Fig. S1. Forecasting performance using finalized data. Compare to Figure 3 in the manuscript.

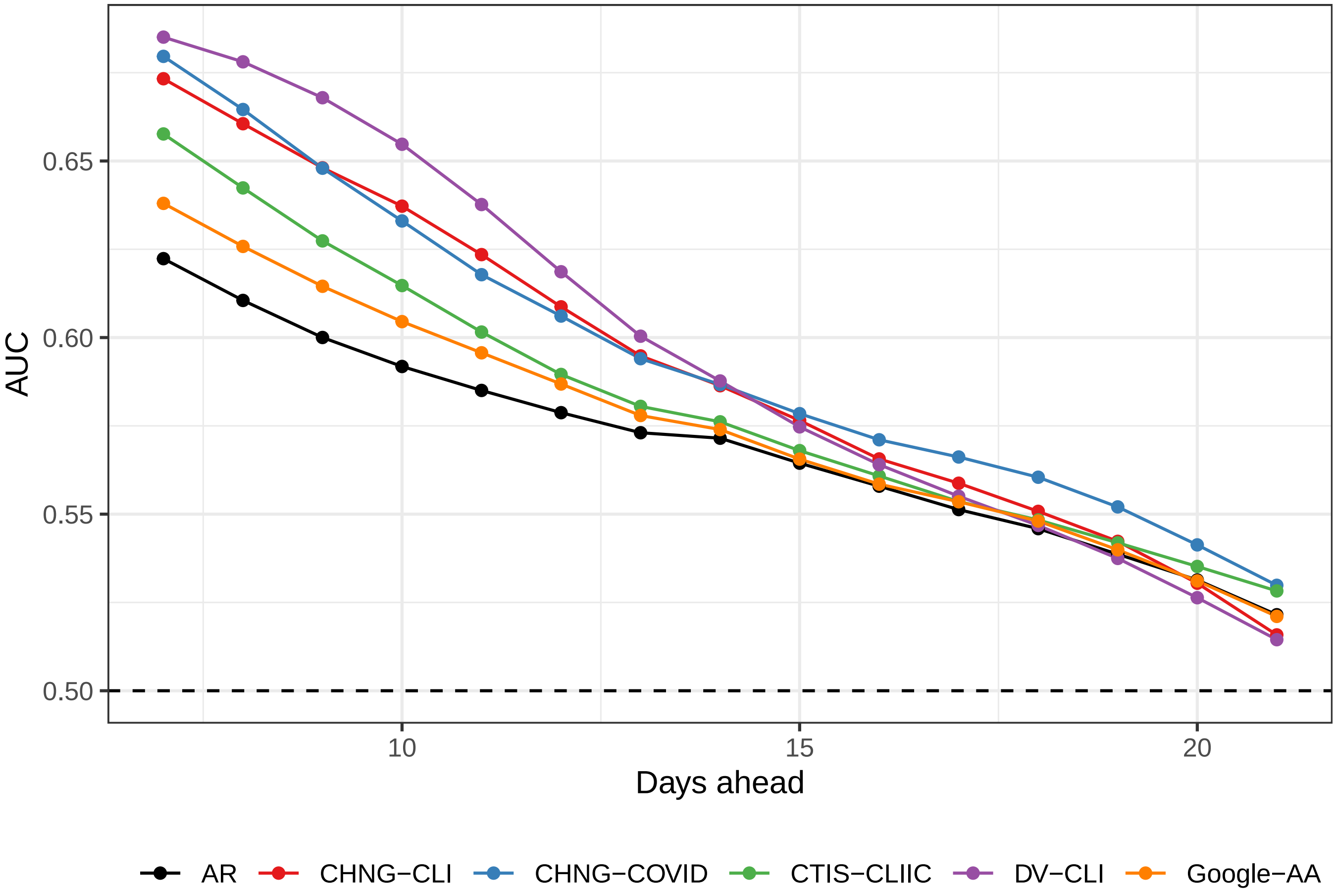

Fig. S2. Hotspot prediction performance using finalized data. Compare to Figure 4 in the manuscript.

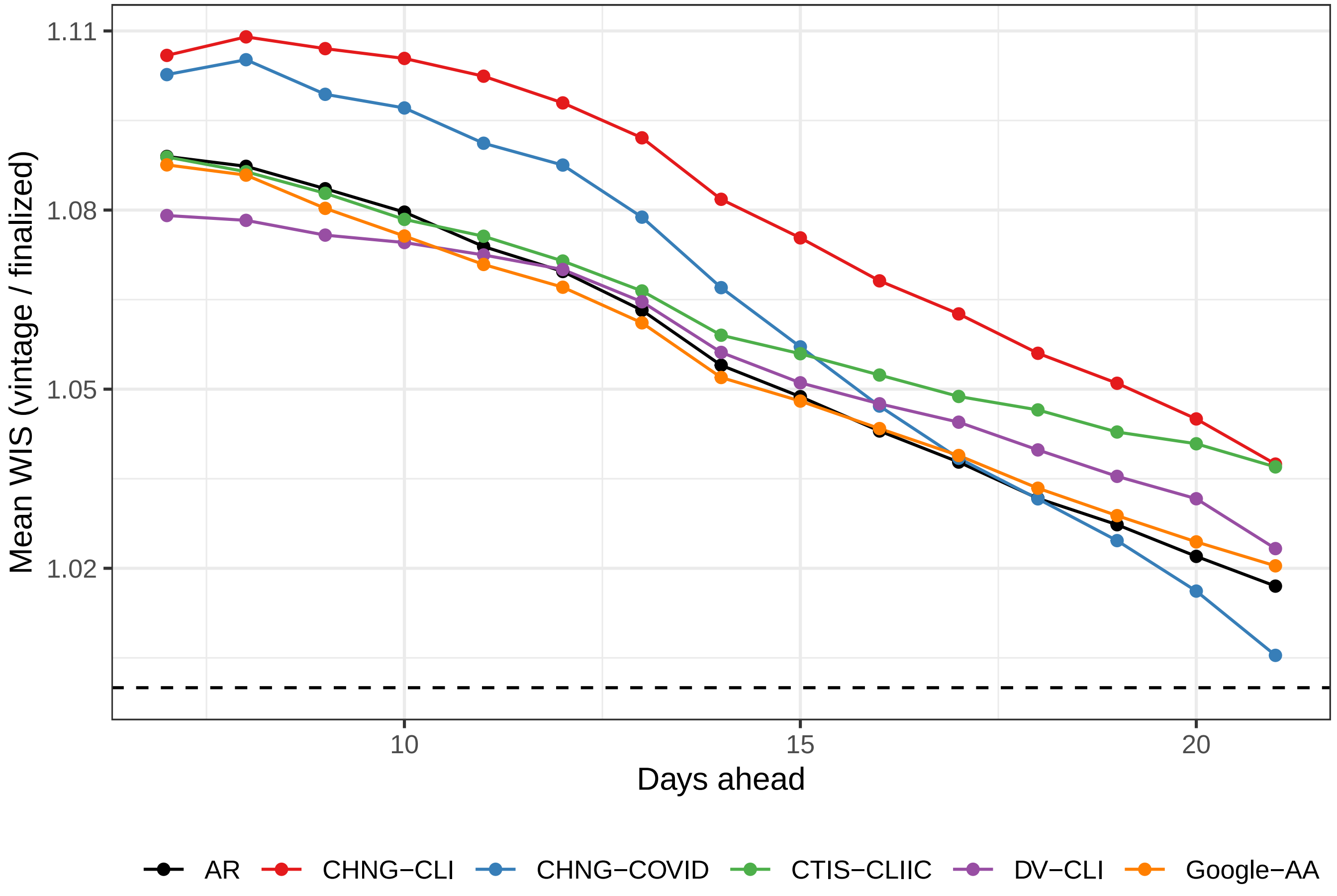

Fig. S3. Forecast performance with vintage compared to finalized data. Using finalized data leads to overly optimistic performance.

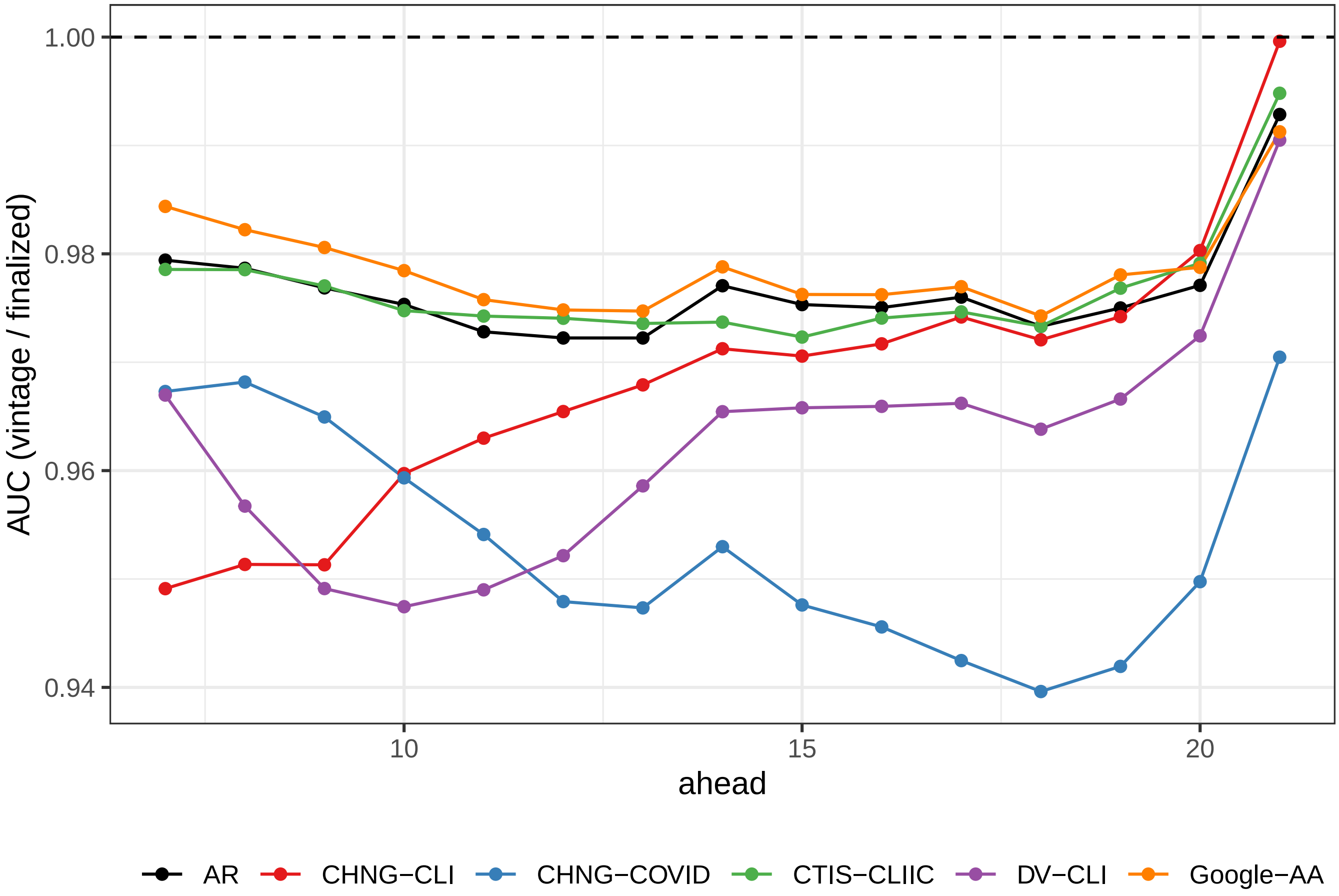

Fig. S4. Hotspot prediction performance with vintage compared to finalized data. Using finalized data leads to overly optimistic performance.

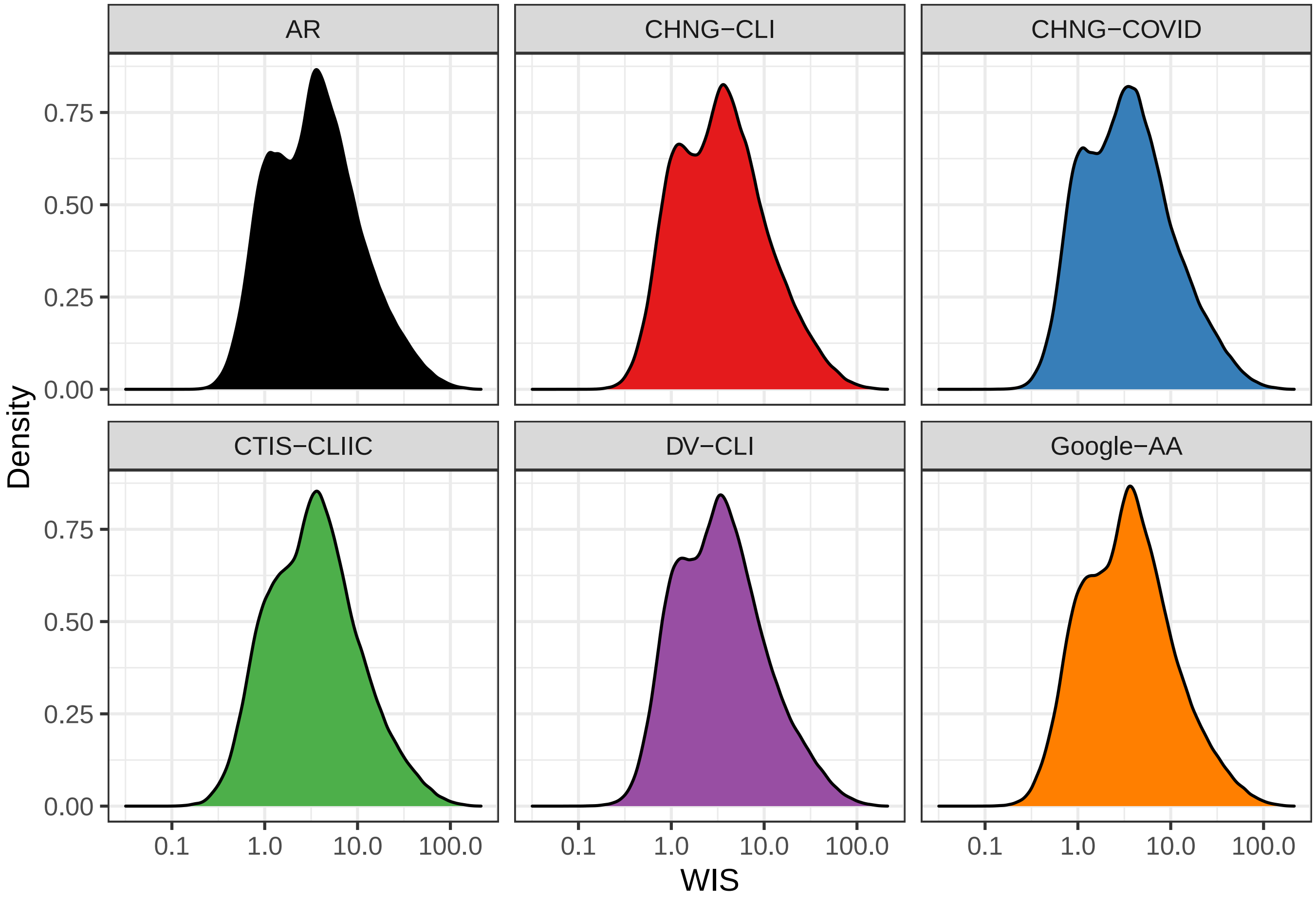

Fig. S5. WIS values from forecast models, which appear to be roughly log-Gaussian.

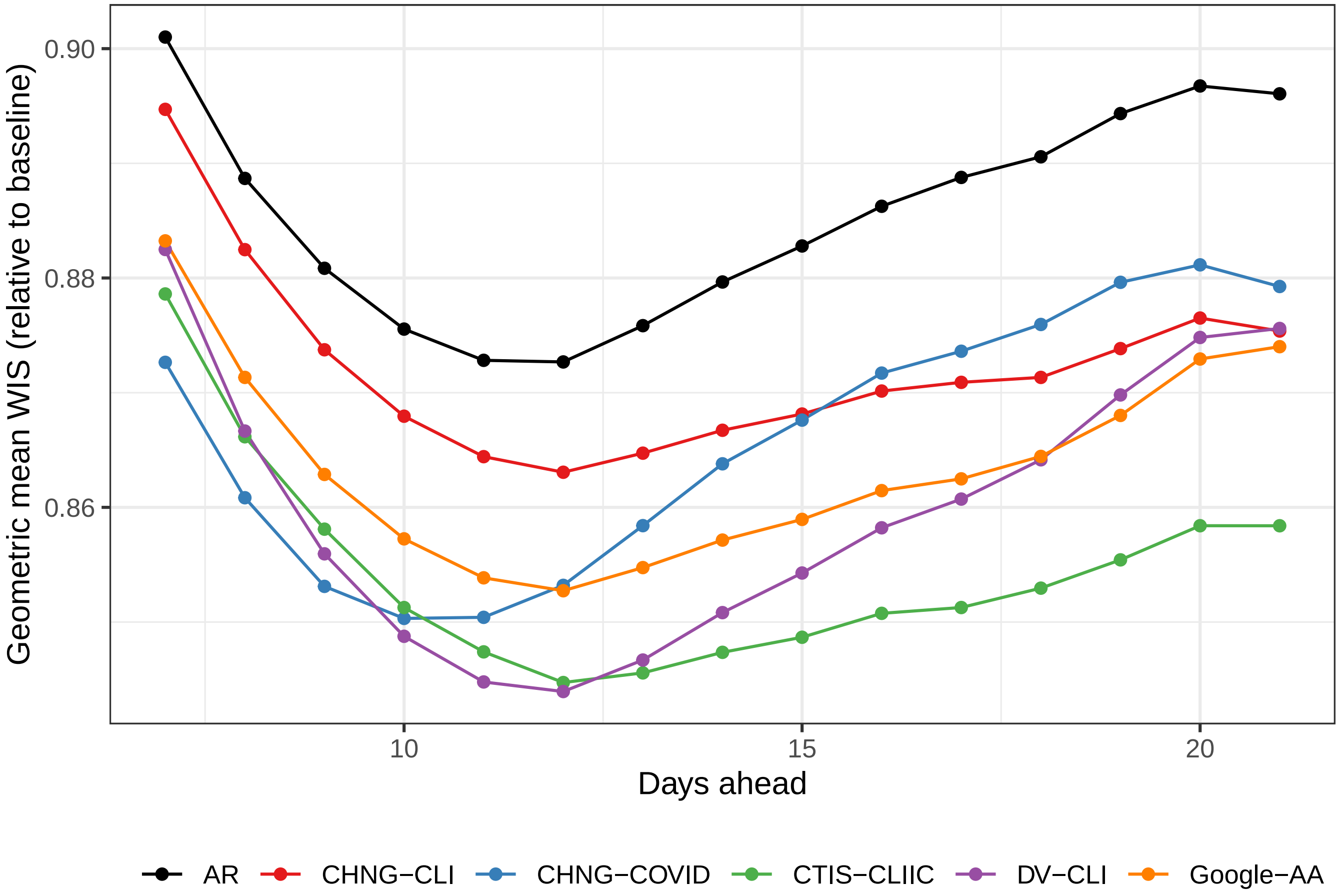

Fig. S6. Forecast performance (using vintage data), summarized by geometric mean.

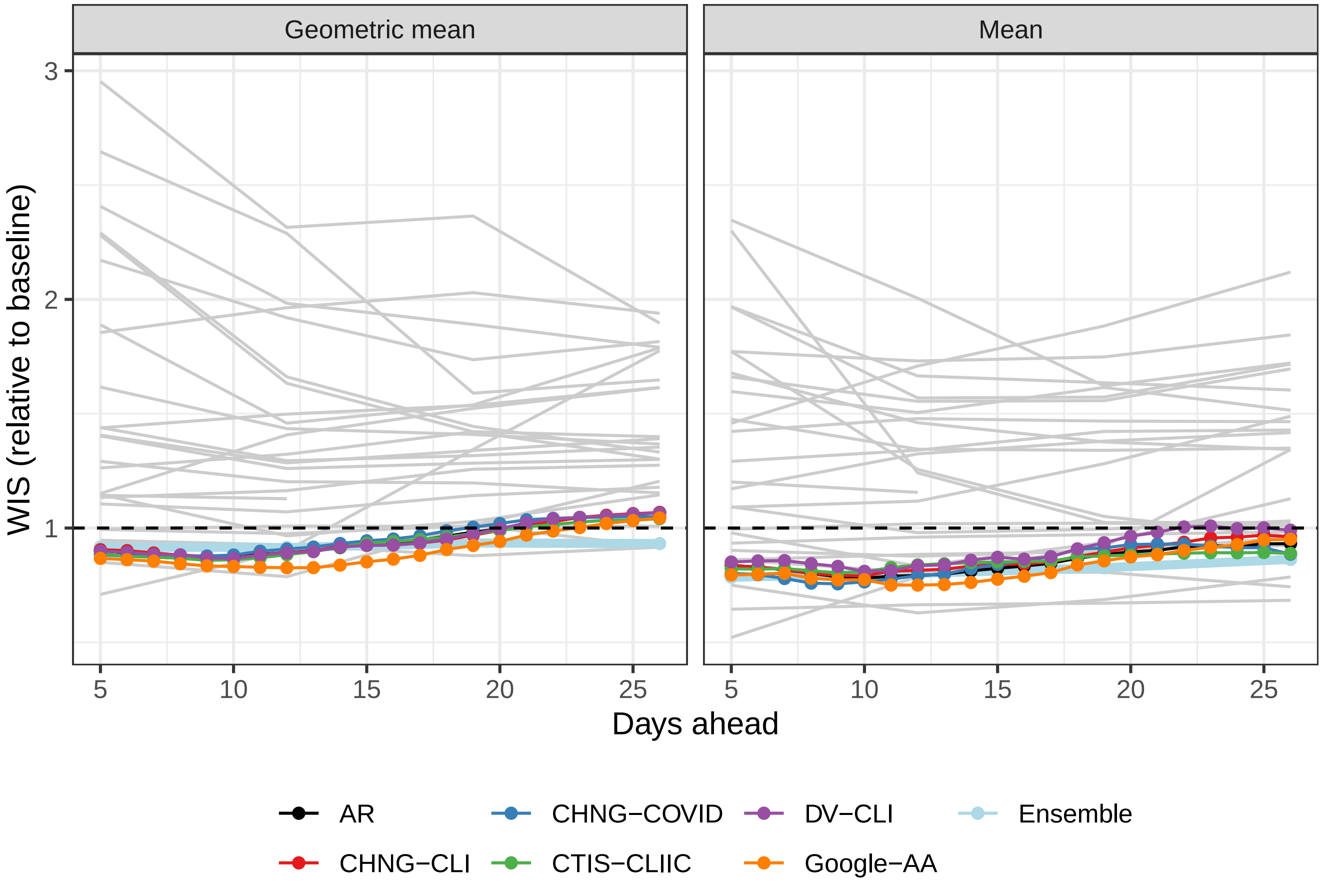

Fig. S7. Forecast performance for AR and indicator models, each retrained at the state level, compared to models submitted to the COVID-19 Forecast Hub over the same period. The thin grey lines are individual models from the Hub; the light blue line is the Hub ensemble model. To align prediction dates as best as possible, we look at the AR and indicator model forecasts for 5, 12, 19, and 26 days ahead; this roughly corresponds to 1, 2, 3, and 4 weeks ahead, respectively, since in the Hub, models typically submit forecasts on a Tuesday for the epiweeks aligned to end on each of the following 4 Saturdays.

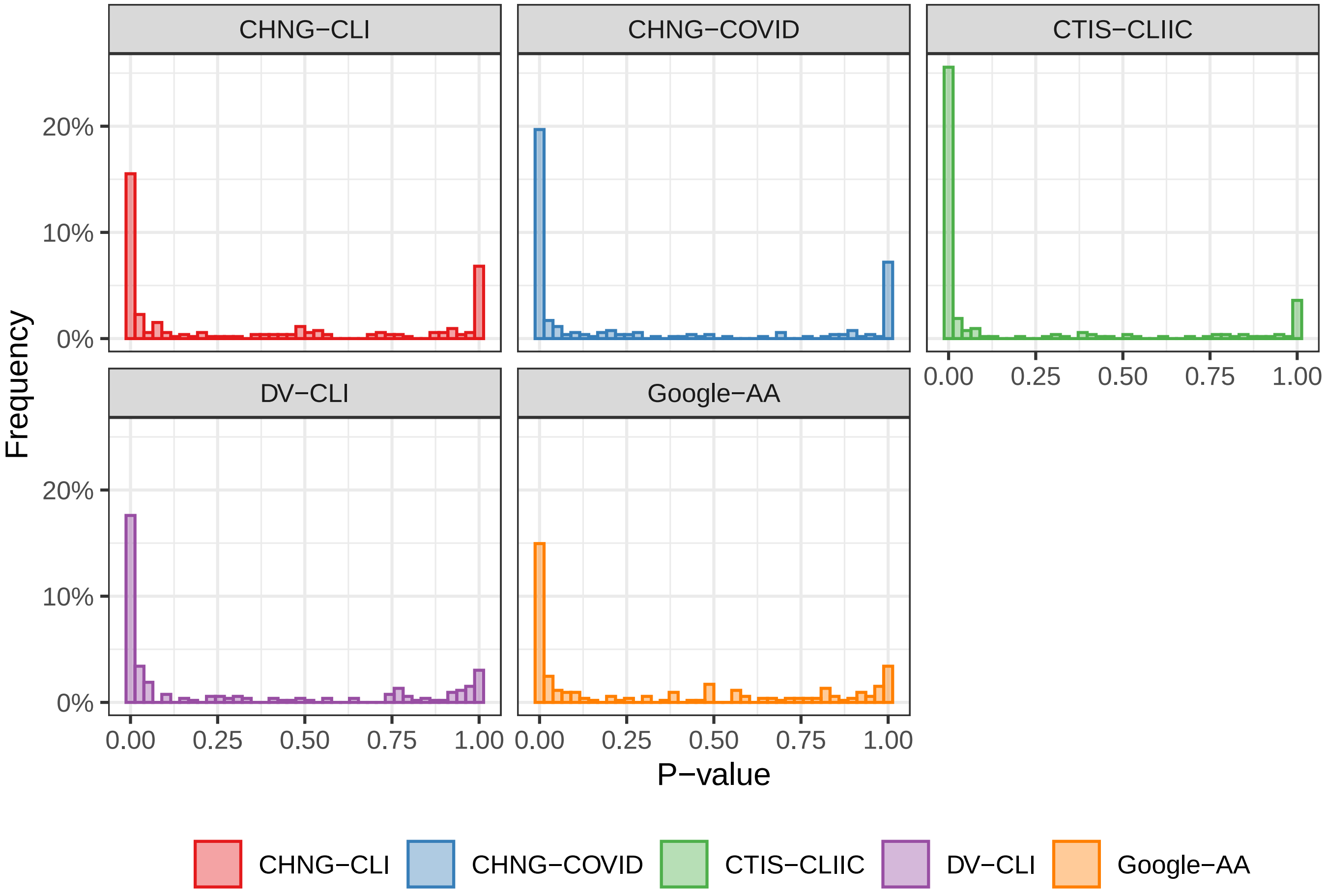

Fig. S8. P-values from a one-sided sign test for improved forecast performance of the indicator-assisted models. Each p-value corresponds to a forecast date. The alternative hypothesis is that the AR model is better (median difference between the relative WIS of the AR model and an indicator model is negative).

Table S1. P-values from a one-sided Diebold-Mariano test for improvemed forecast performance when adding the indicators. The alternative hypothesis is that the AR model is better.

metric	CHNG-CLI	CHNG-COVID	CTIS-CLIIC	DV-CLI	Google-AA
Geometric mean	0.072	0.036	0.005	0.032	0.026
Mean	0.177	0.132	0.006	0.092	0.103

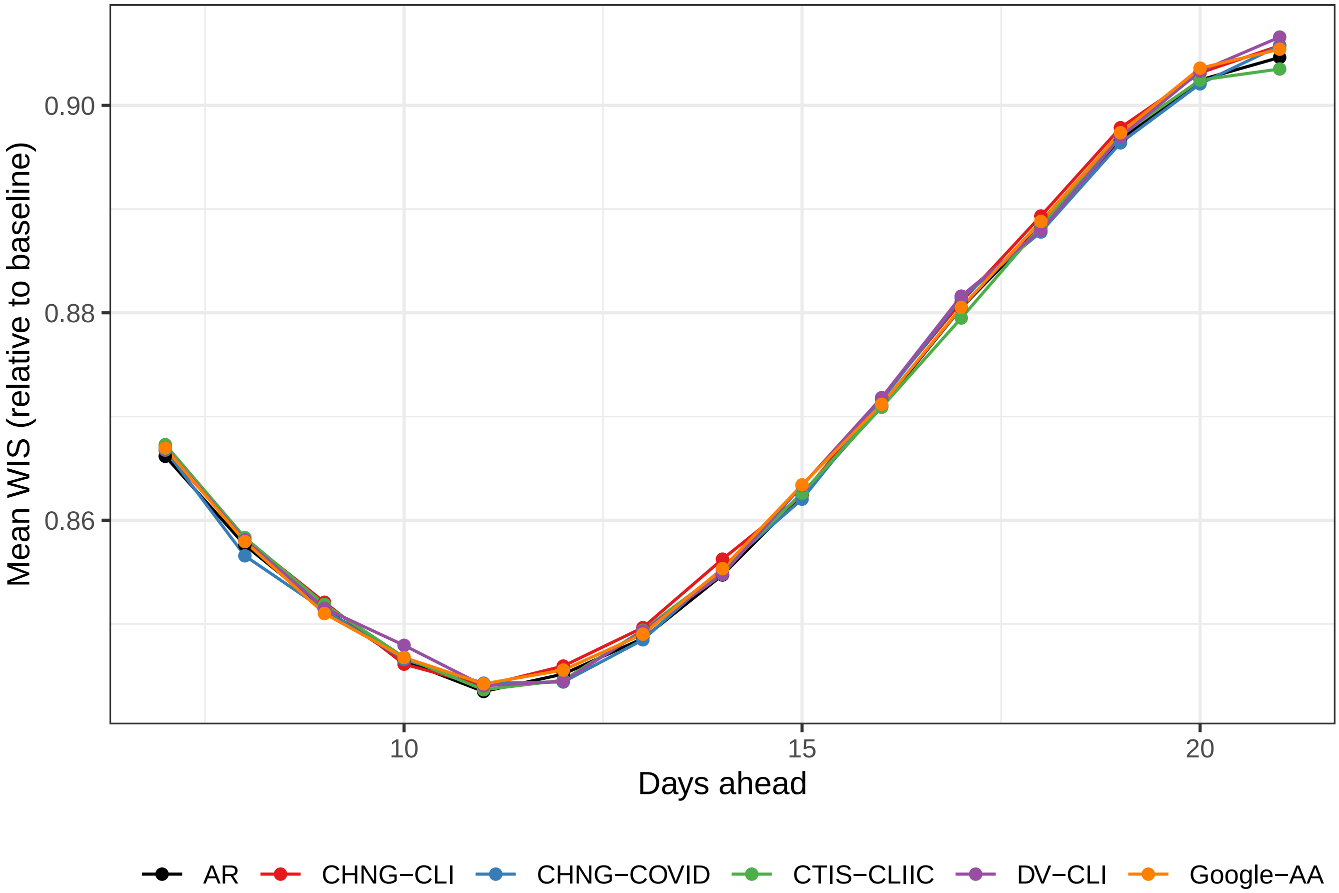

Fig. S9. Forecast performance when indicators are replaced with samples from their empirical distribution. Performance is largely similar to the AR model.

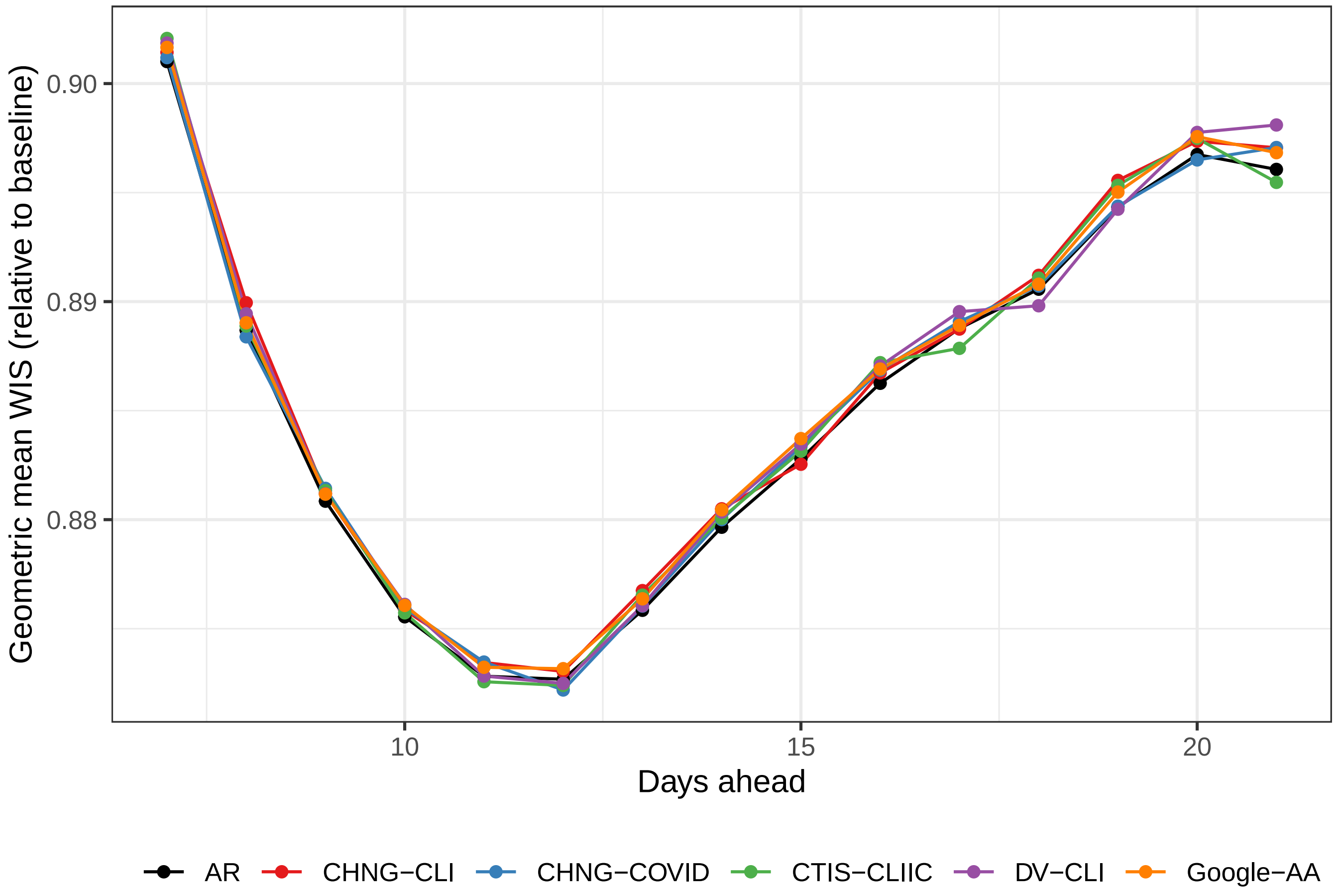

Fig. S10. Forecast performance as measured with the geometric mean when indicators are replaced with samples from their empirical distribution. Performance is largely similar to the AR model.

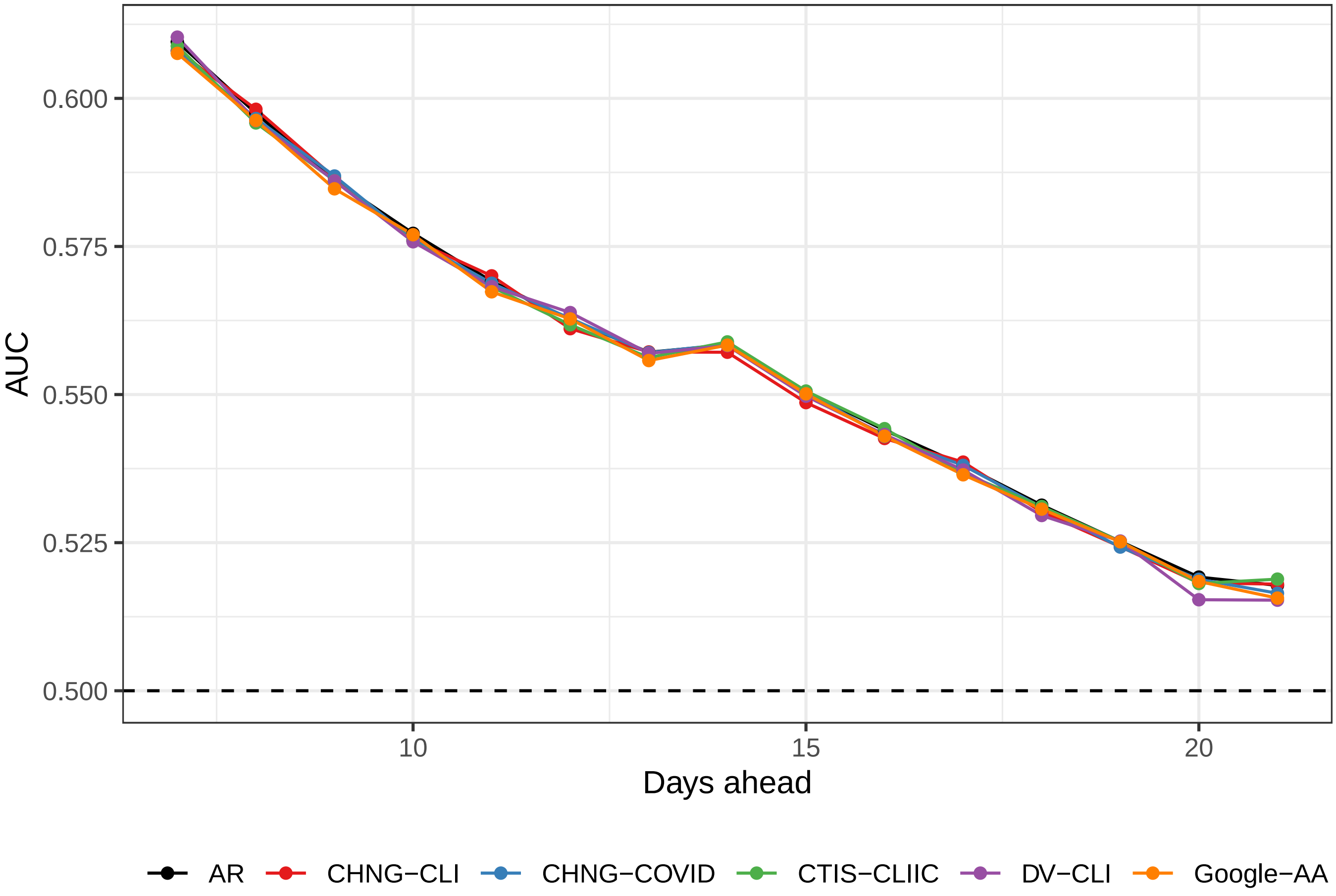

Fig. S11. Hotspot prediction performance when indicators are replaced with samples from their empirical distribution. Performance is largely similar to the AR model.

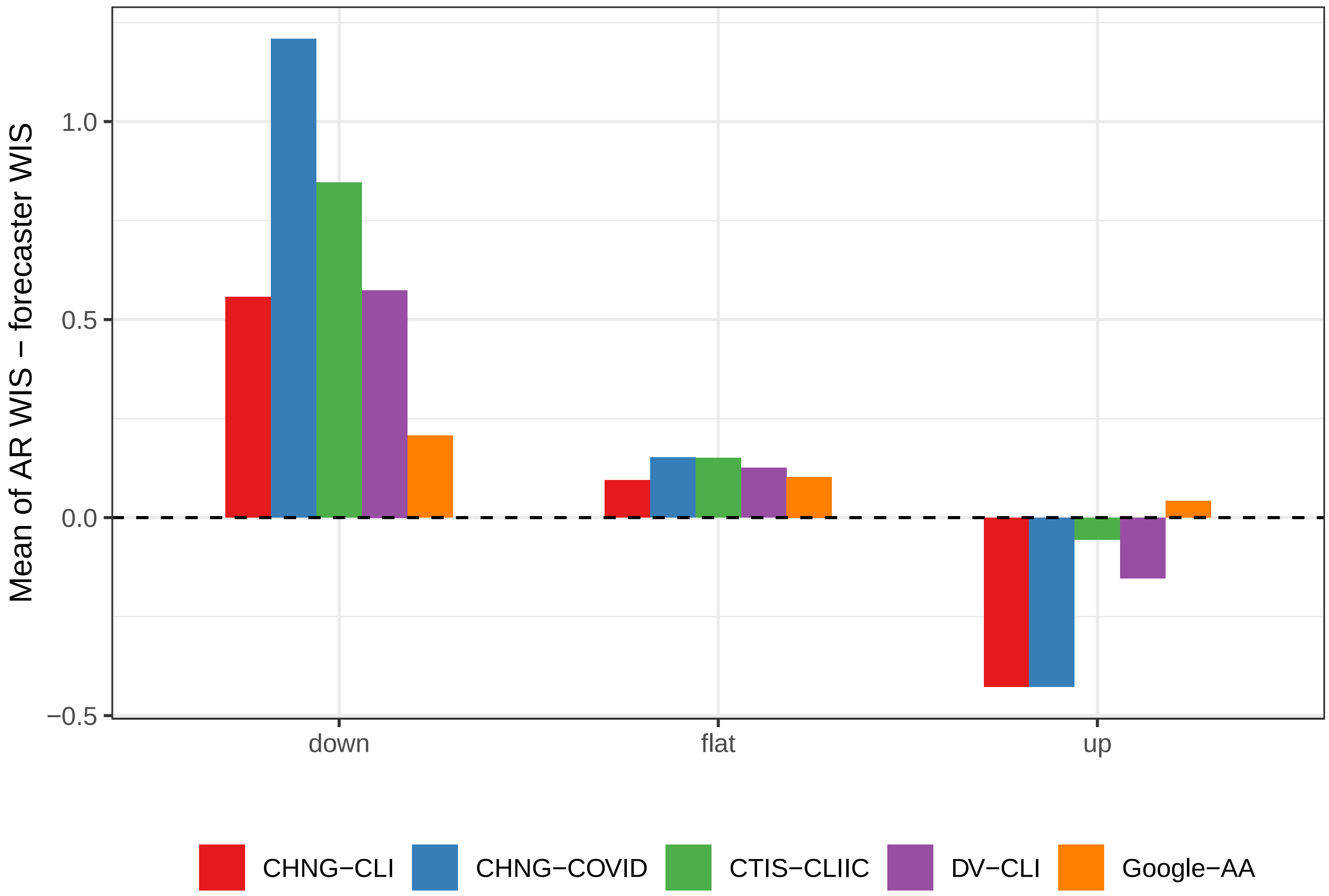

Fig. S12. Average difference between the WIS of the AR model and of the indicator models, separated into up, down, and flat periods. The indicator models generally do best during down and flat periods.

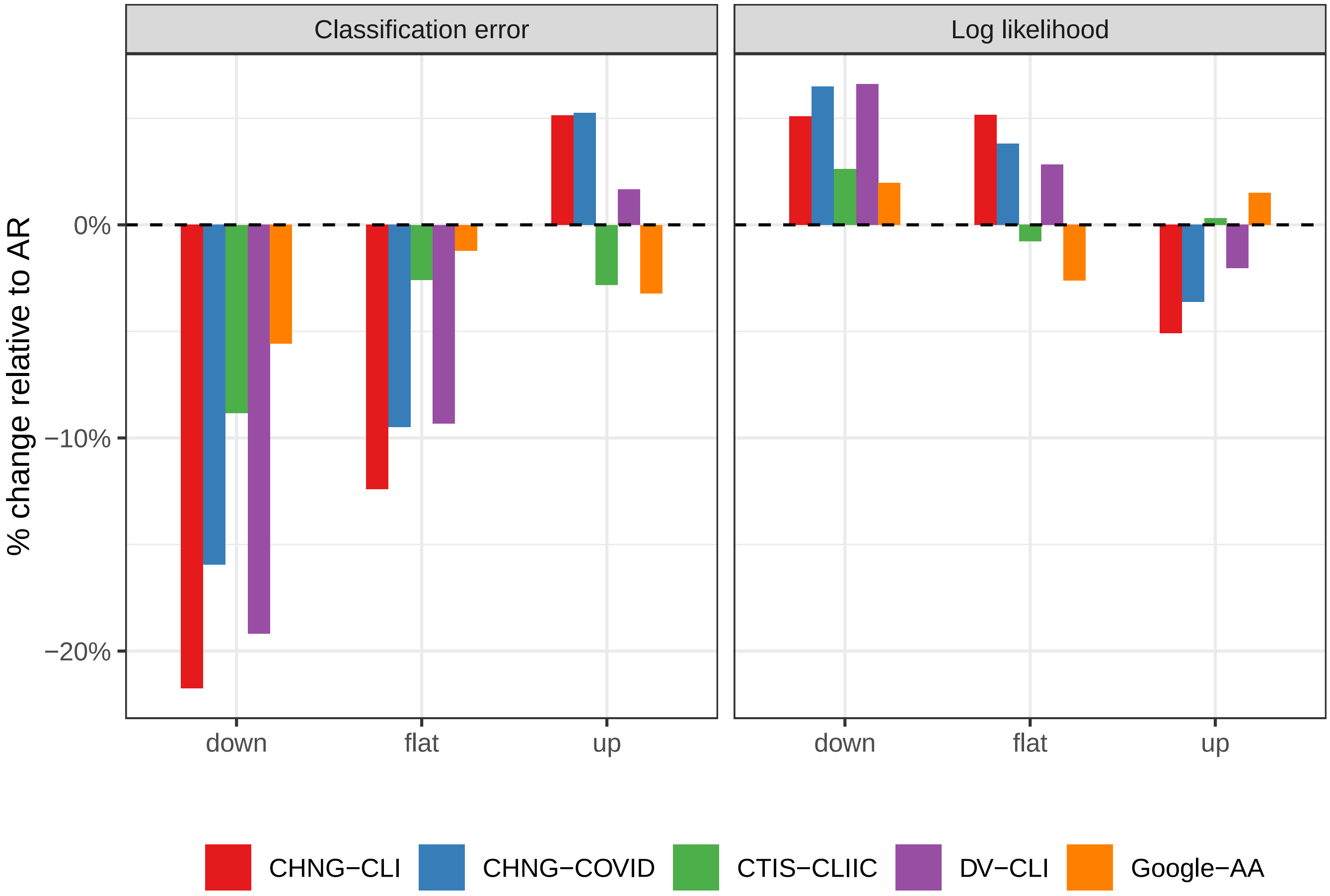

Fig. S13. Percentage change in classification error and log likelihood, relative that of the AR model, separated into up, down, and flat periods. Like the analogous forecasting analysis, the indicator models generally do better during down and flat periods.

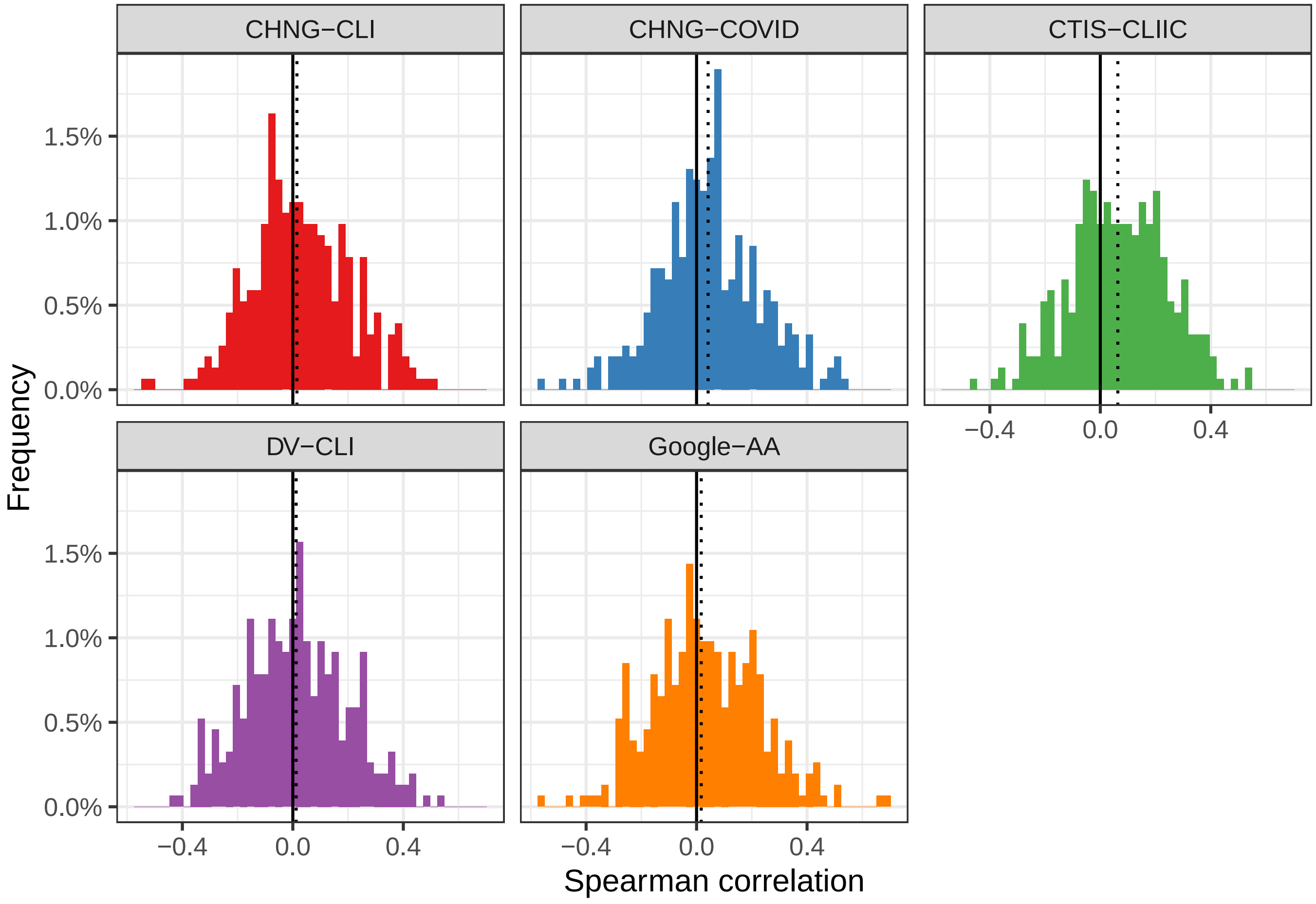

Fig. S14. Histograms of the Spearman correlation between the ratio of forecaster WIS to AR WIS with the % change in case rates, relative to case rates 7 days earlier.

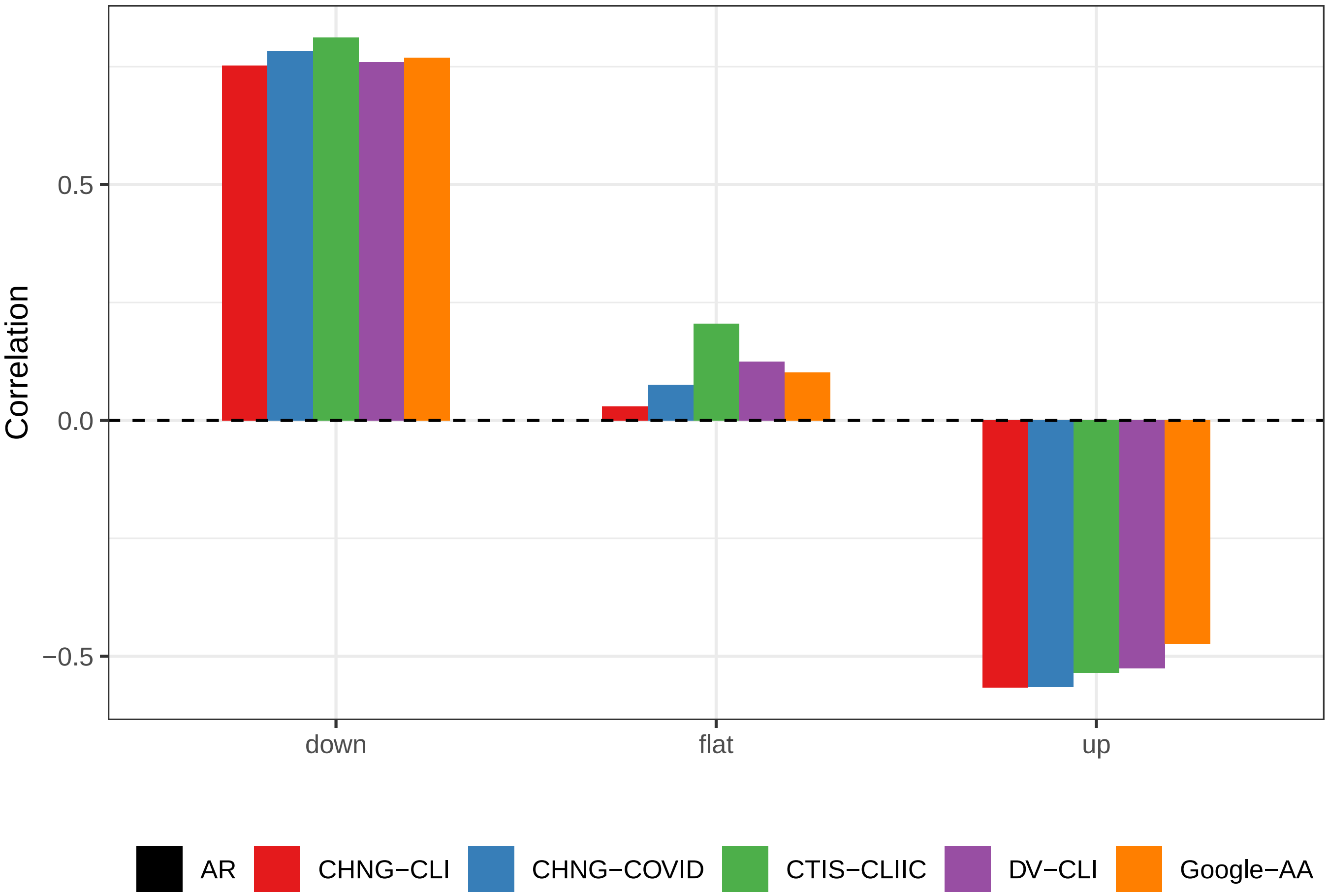

Fig. S15. Correlation of the difference in WIS with the difference in median predictions (each difference being between the AR model and an indicator model), separated into up, down, and flat periods. In down periods, improvements in forecast error are highly correlated with lower median predictions. The opposite is true in up periods, but the conclusion here appears to be weaker.

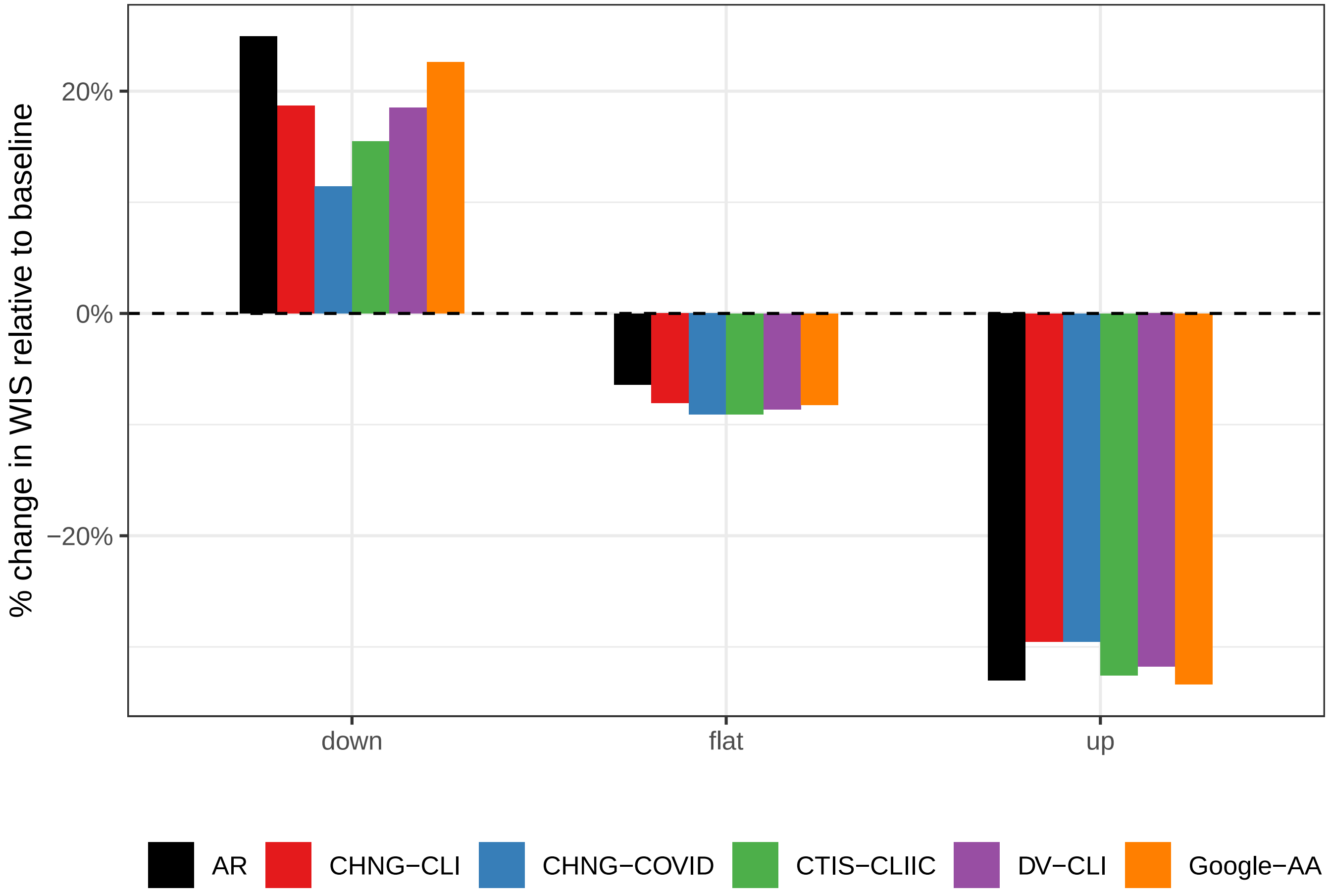

Fig. S16. Percentage change in average WIS of the forecaster (AR or indicator assisted), relative to the baseline. All models perform poorly during down periods, but the indicators help. During flat periods, the indicators improve slightly over the AR. During up periods, all forecasters do much better than the baseline, but only some do as well as AR.

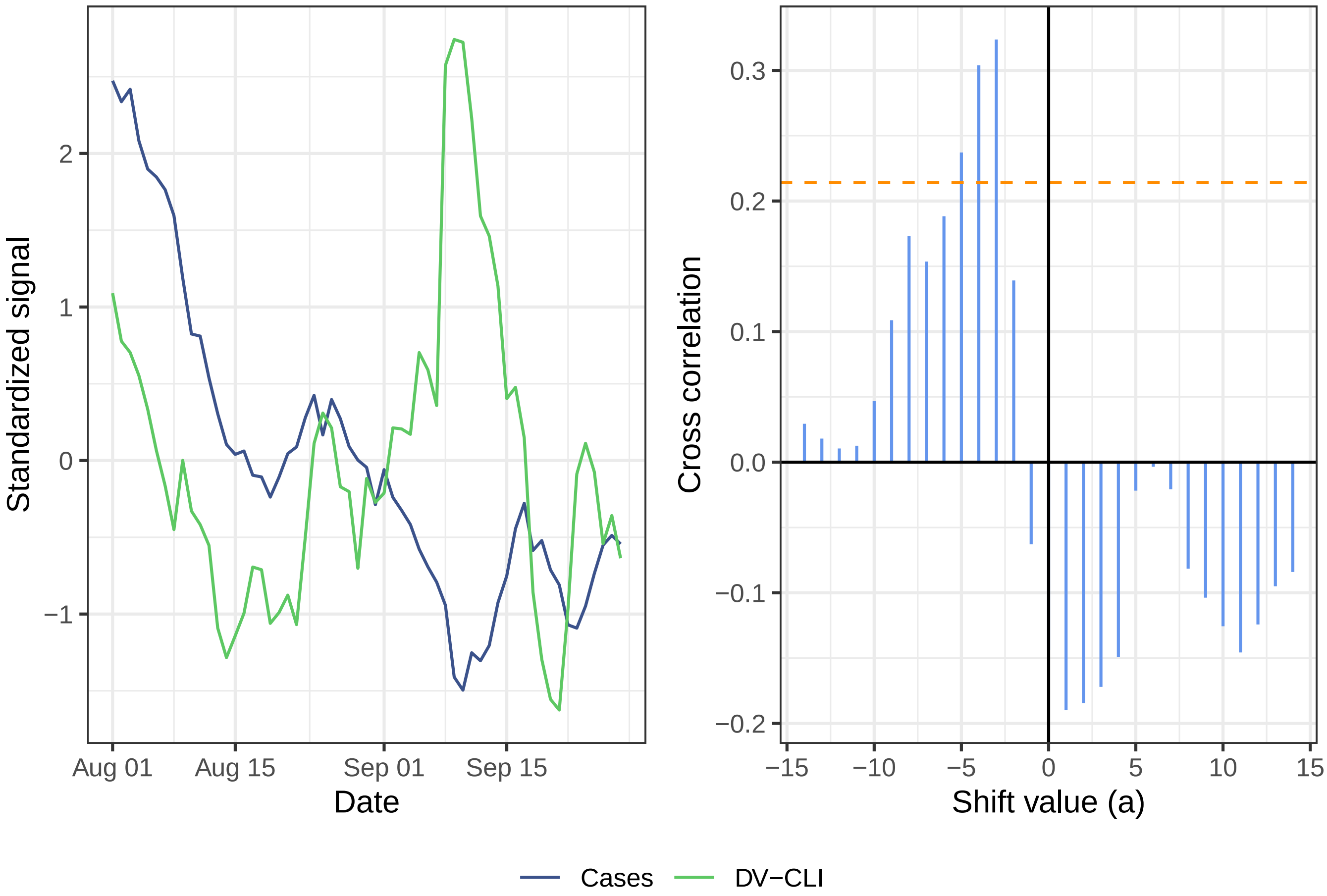

Fig. S17. Illustration of the cross-correlation function between DV-CLI and cases. The left panel shows the standardized signals over the period from August 1 to September 28 (as of May 15, 2021). The right panel shows $\mathrm{CCF}_\ell(a)$ for different values of a as vertical blue bars. The orange dashed line indicates the 95% significance threshold. By our leadingness/laggingness metric, DV-CLI is leading (but not lagging) cases over this period.

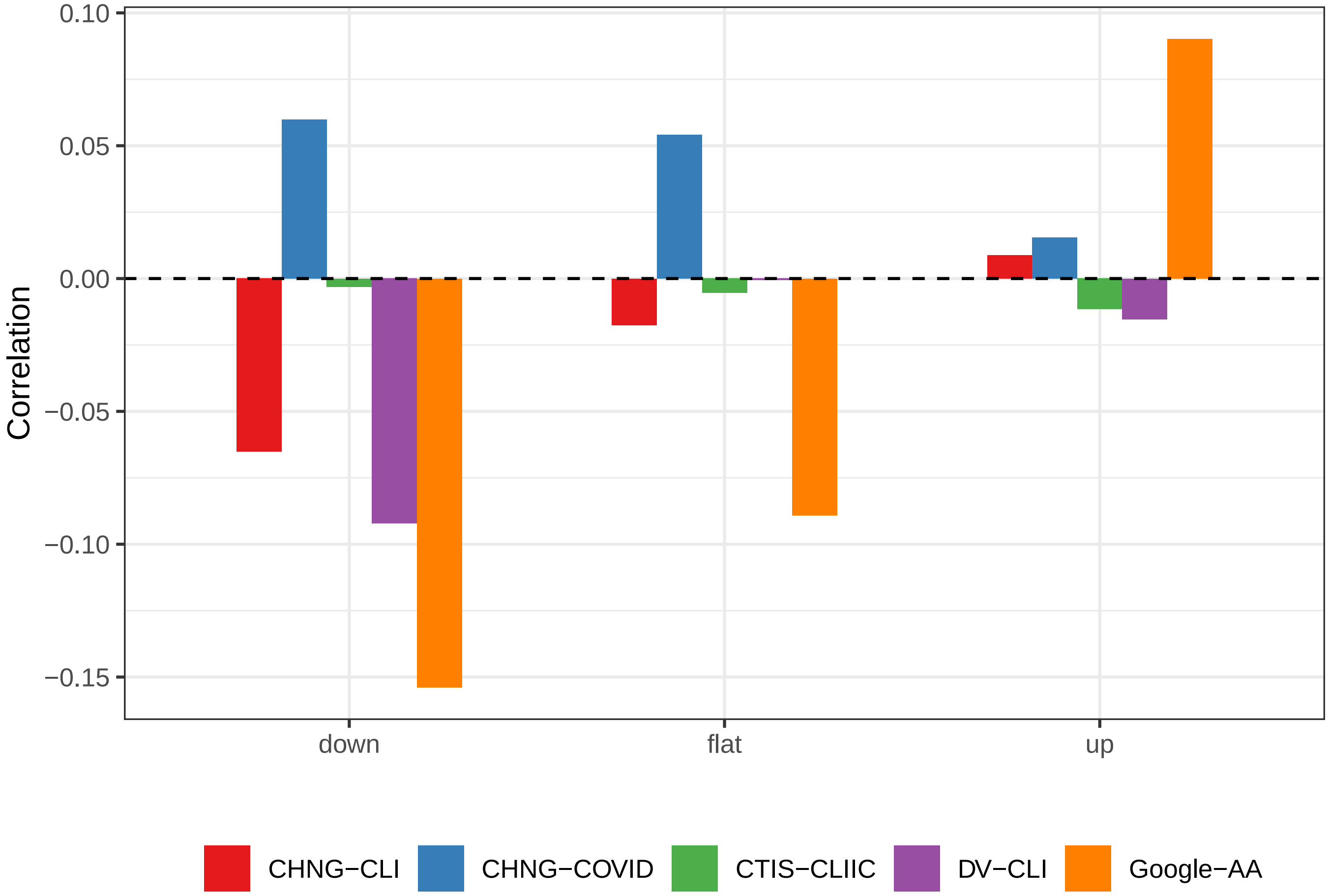

Fig. S18. Correlation of the difference in WIS with the laggingness of the indicator at the target date, stratified by up, down, or flat period. Compare to Figure 5 in the manuscript.

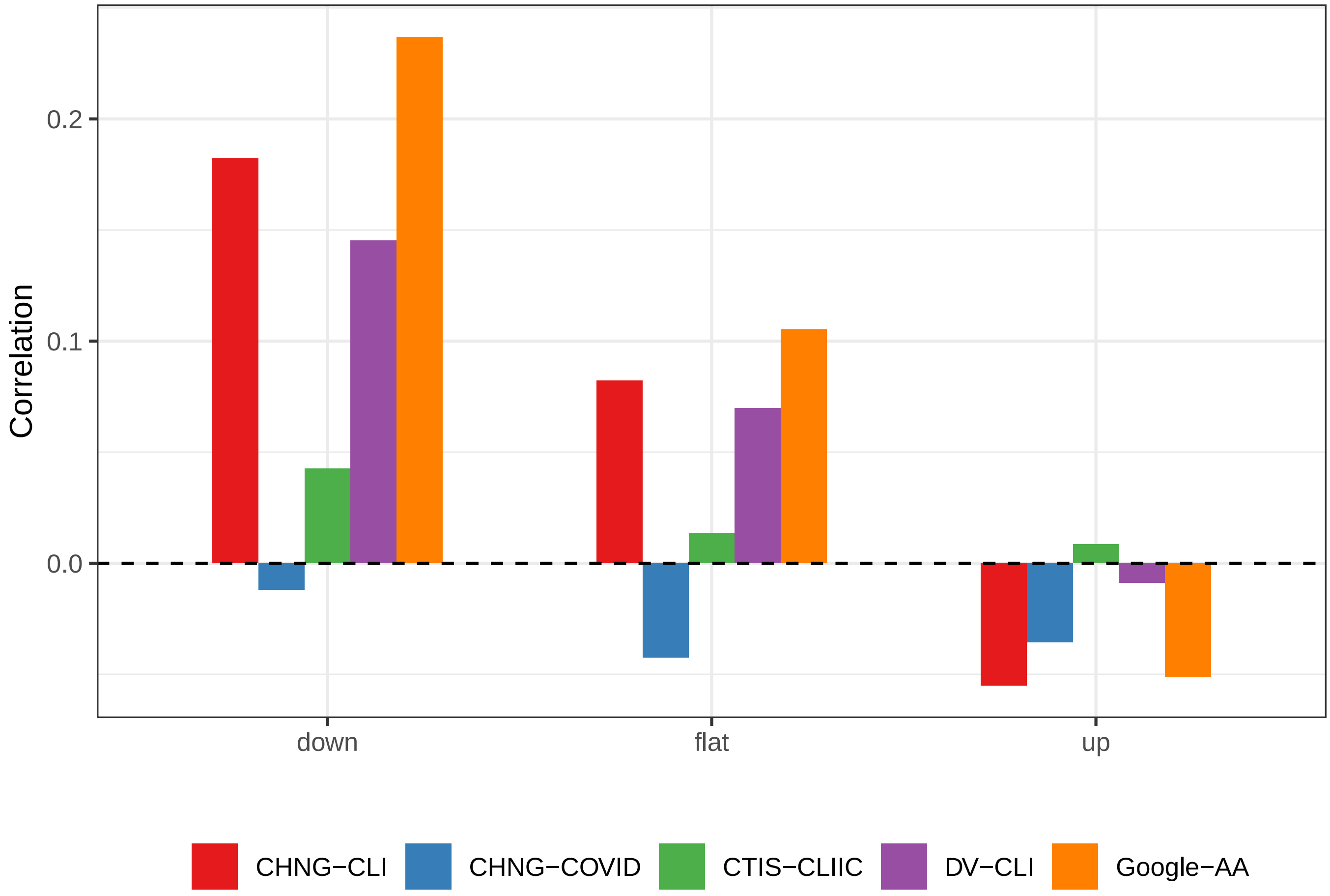

Fig. S19. Correlation of the difference between leadingness and laggingness with the difference in WIS. The relationship is essentially the same as described in the manuscript and shown in Figure 5.

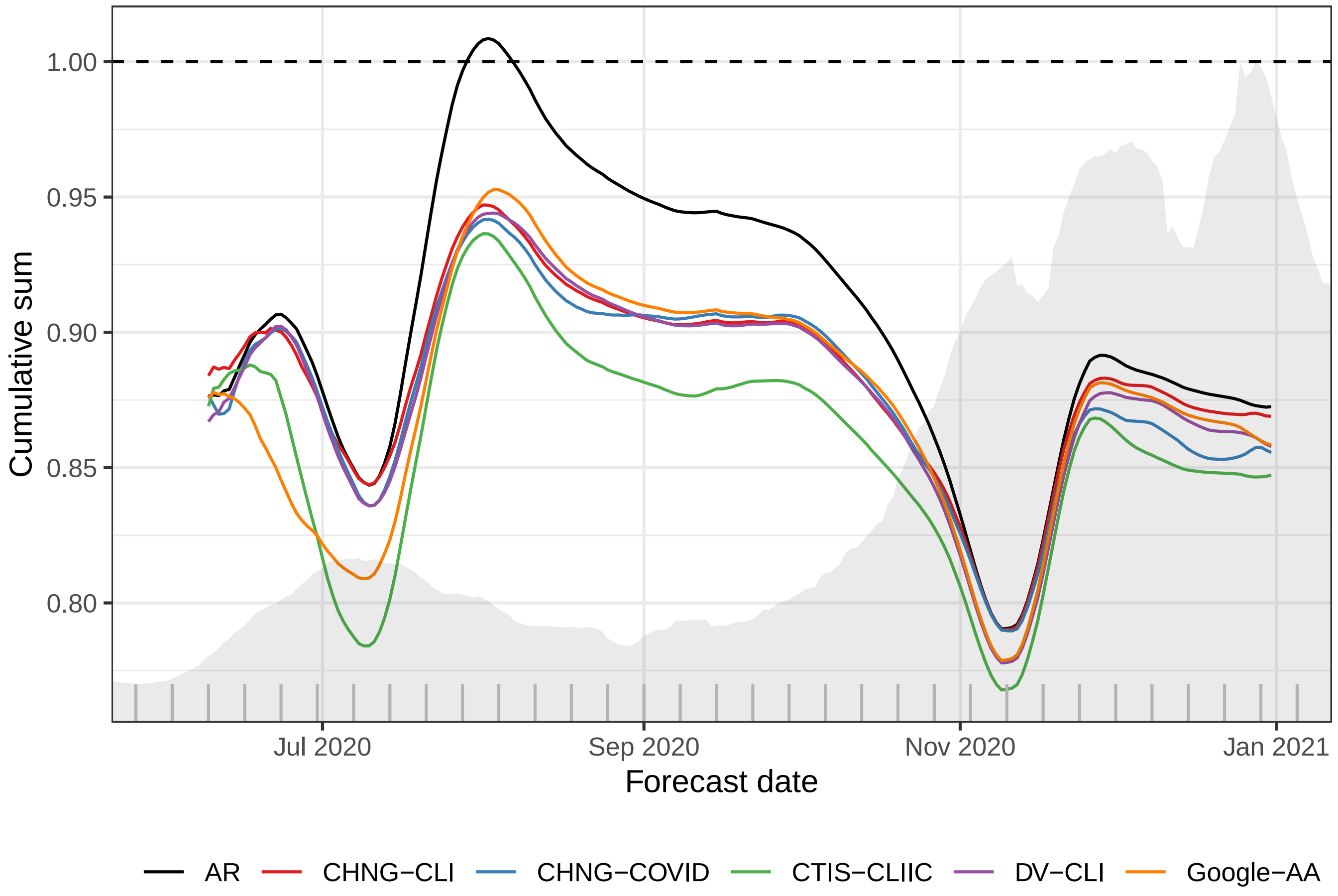

Fig. S20. Cumulative sum of WIS for each forecaster divided by the cumulative sum of WIS for the baseline model. The shaded background shows national case incidence for the 14-day ahead target. Hash marks along the x-axis denote weeks.

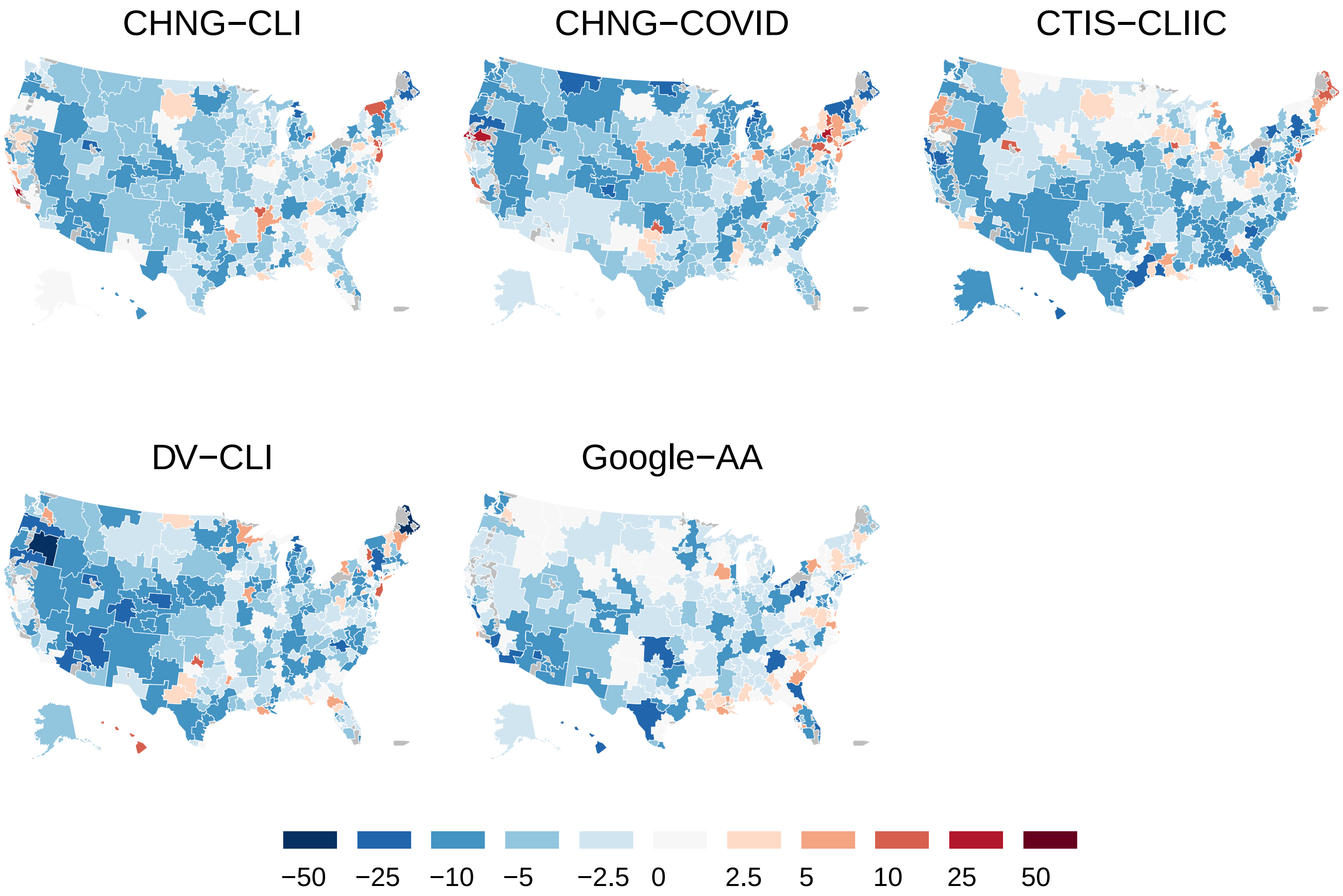

Fig. S21. Percentage improvement in WIS, relative to the AR forecaster, by HRR (negative numbers indicate improved performance, positives indicate worsening).

SI Dataset S1 (Code)

Code for reproducibility. `R` script files and small datasets. Persistent DOI: https://doi.org/10.5281/zenodo.5639567

SI Dataset S2 (Data)

Large intermediate data sets. Archived `.RDS` (R objects) files containing all evaluations for forecasting and hotspots using vintage and finalized data. Can be used to produce the graphics and conclusions in the manuscript without rerunning the entire pipeline. Persistent DOI: https://doi.org/10.5683/SP3/UW4VTC

References

1 Reich Lab, The COVID-19 Forecast Hub (https://covid19forecasthub.org) (2020).
2 FX Diebold, RS Mariano, Comparing predictive accuracy. *J. Bus. & Econ. Stat.* **20**, 134–144 (2002).
3 FX Diebold, Comparing predictive accuracy, twenty years later: A personal perspective on the use and abuse of Diebold–Mariano tests. *J. Bus. & Econ. Stat.* **33**, 1–24 (2015).
4 D Harvey, S Leybourne, P Newbold, Testing the equality of prediction mean squared errors. *Int. J. Forecast.* **13**, 281–291 (1997).
